# Prevalence of *BRCA1*, *BRCA2*, and *PALB2* genomic alterations among 924 Taiwanese breast cancer assays with tumor-only targeted sequencing: extended data analysis from the VGH-TAYLOR study

**DOI:** 10.1186/s13058-023-01751-z

**Published:** 2023-12-14

**Authors:** Han-Fang Cheng, Yi-Fang Tsai, Chun-Yu Liu, Chih-Yi Hsu, Pei-Ju Lien, Yen-Shu Lin, Ta-Chung Chao, Jiun-I. Lai, Chin-Jung Feng, Yen-Jen Chen, Bo-Fang Chen, Jen-Hwey Chiu, Ling-Ming Tseng, Chi-Cheng Huang

**Affiliations:** 1 Comprehensive Breast Health Center, Department of Surgery, Taipei Veterans General Hospital, Taipei City, Taiwan, ROC; 2 School of Medicine, College of Medicine, National Yang Ming Chiao Tung University, Taipei City, Taiwan, ROC; 3 Division of Transfusion Medicine, Department of Medicine, Taipei Veterans General Hospital, Taipei City, Taiwan, ROC; 4 Division of Medical Oncology, Department of Oncology, Taipei Veterans General Hospital, Taipei City, Taiwan, ROC; 5 Department of Pathology and Laboratory Medicine, Taipei Veterans General Hospital, Taipei City, Taiwan, ROC; 6 Department of Nurse, Taipei Veterans General Hospital, Taipei City, Taiwan, ROC; 7 Institute of Clinical Medicine, National Yang Ming Chiao Tung University, Taipei City, Taiwan, ROC; 8 Division of Plastic and Reconstruction Surgery, Department of Surgery, Taipei Veterans General Hospital, Taipei City, Taiwan, ROC; 9 Center for Traditional Medicine, Taipei Veterans General Hospital, Taipei City, Taiwan, ROC; 10 Institue of Traditional Medicine, College of Medicine, National Yang Ming Chiao Tung University, Taipei City, Taiwan, ROC; 11 Department of Surgery, Taipei Veterans General Hospital, Taipei City, Taiwan, ROC; 12 Institute of Epidemiology and Preventive Medicine, College of Medicine, National Taiwan University, Taipei City, Taiwan, ROC

**Keywords:** *BRCA1/2*, *PALB2*, Tumor-only targeted sequencing, Breast cancer, Taiwan

## Abstract

**Background:**

The homologous recombination (HR) repair pathway for DNA damage, particularly the *BRCA1* and *BRCA2* genes, has become a target for cancer therapy, with poly ADP-ribose polymerase (PARP) inhibitors showing significant outcomes in treating germline *BRCA1/2* (g*BRCA1/2*) mutated breast cancer. Recent studies suggest that some patients with somatic *BRCA1/2* (s*BRCA1/2*) mutation or mutations in HR-related genes other than *BRCA1/2* may benefit from PARP inhibitors as well, particularly those with *PALB2* mutations. The current analysis aims to evaluate the prevalence of genetic alterations specific to *BRCA1*, *BRCA2*, and *PALB2* in a large cohort of Taiwanese breast cancer patients through tumor-targeted sequencing.

**Methods:**

A total of 924 consecutive assays from 879 Taiwanese breast cancer patients underwent tumor-targeted sequencing (Thermo Fisher Oncomine Comprehensive Assay v3). We evaluated *BRCA1*, *BRCA2*, and *PALB2* mutational profiles, with variants annotated and curated by the ClinVAR, the Oncomine™ Knowledgebase Reporter, and the OncoKB™. We also conducted reflex germline testing using either whole exome sequencing (WES) or whole genome sequencing (WGS), which is ongoing.

**Results:**

Among the 879 patients analyzed (924 assays), 130 had positive mutations in *BRCA1* (3.1%), *BRCA2* (8.6%), and *PALB2* (5.2%), with a total of 14.8% having genetic alterations. Co-occurrence was noted between *BRCA1/BRCA2*, *BRCA1/PALB2,* and *BRCA2/PALB2* mutations. In *BRCA1*-mutated samples, only p.K654fs was observed in three patients, while other variants were observed no more than twice. For *BRCA2*, p.N372H was the most common (26 patients), followed by p.S2186fs, p.V2466A, and p.X159_splice (5 times each). For *PALB2*, p.I887fs was the most common mutation (30 patients). This study identified 176 amino acid changes; 60.2% (106) were not documented in either ClinVAR or the Oncomine™ Knowledgebase Reporter. Using the OncoKB™ for annotation, 171 (97.2%) were found to have clinical implications. For the result of reflex germline testing, three variants (*BRCA1* c.1969_1970del, *BRCA1* c.3629_3630del, *BRCA2* c.8755-1G > C) were annotated as Pathogenic/Likely pathogenic (P/LP) variants by ClinVar and as likely loss-of-function or likely oncogenic by OncoKB; while one variant (*PALB2* c.448C > T) was not found in ClinVar but was annotated as likely loss-of-function or likely oncogenic by OncoKB.

**Conclusion:**

Our study depicted the mutational patterns of *BRCA1*, *BRCA2*, and *PALB2* in Taiwanese breast cancer patients through tumor-only sequencing. This highlights the growing importance of *BRCA1/2* and *PALB2* alterations in breast cancer susceptibility risk and the treatment of index patients. We also emphasized the need to meticulously annotate variants in cancer-driver genes as well as actionable mutations across multiple databases.

**Supplementary Information:**

The online version contains supplementary material available at 10.1186/s13058-023-01751-z.

## Background

Since the discovery of the *BRCA1* and *BRCA*2 genes in 1994 and 1995 [1, 2], the homologous recombination (HR) repair pathway for DNA damage has become a focus area for tumorigenesis and cancer therapy. Preclinical studies in 2005 demonstrated that poly ADP-ribose polymerase inhibitors (PARP) inhibitors selectively target *BRCA*-deficient cells, a decade after the discovery of the *BRCA* genes [3]. Nowadays, numerous phase III clinical trials have shown that various PARP inhibitors can improve treatment outcomes in both early and advanced germline *BRCA1/2* (g*BRCA1/2*)-mutant breast cancer, as well as achieve a better quality of life [4–7], opposed to cytotoxic chemotherapy.

Although PARP inhibitors have shown tremendous treatment outcomes in prolonging progression-free survival for g*BRCA1/2*-mutant breast cancer and extending overall survival for high-risk early-stage cancer, the prevalence of these mutations is estimated to be only 2–5% in unselected general population [8–11]. Therefore, it is important to identify patients beyond g*BRCA1/2* carriers whose cancers may be sensitive to PARP inhibition, given the limited population of g*BRCA1/2* mutations in breast cancer patients.

Studies in prostate and ovarian cancer have suggested that some patients with somatic *BRCA1/2* (s*BRCA1/2*) mutation or mutations in HR-related genes other than *BRCA1/2* may benefit from PARP inhibitors [12–15]. According to the results of the TBCRC 048, an investigator-initiated phase 2 trial to assess Olaparib response in metastatic breast cancer patients with s*BRCA1/2* mutations or germline/somatic mutations in HR-related genes other than *BRCA1/2*, responses were observed only in patients with s*BRCA1/*2 mutations (objective response rate [ORR] 50%) or g*PALB2* mutations (ORR 82%) [16]. The Talazoparib Beyond *BRCA* (TBB) trial included any solid tumor with germline or somatic mutations in HR-related genes other than *BRCA1* and *BRCA2* in cohort B. They reported a 31% overall response rate in breast cancer patients. Furthermore, all five breast cancer patients with g*PALB2* mutations had treatment-associated tumor regression [17]. Among all HR-related genes, *PALB2* is responsible for loading *RAD51* onto ssDNA, stimulating *RAD51*-mediated strand exchange and D-loop formation via the *BRCA* complex (*BRCA1-PALB2-BRCA2-RAD5*1) [18, 19]. Germline *PALB2* mutation is estimated to be present in about 1% of breast cancer patient populations [19–22] and is known for its remarkable increased risk of breast cancer and pancreatic cancer [22]. Better understanding of the patterns regarding both somatic and germline mutations in *BRCA1/2* and *PALB2* can lead to improved treatment outcomes for these specific populations.

The epidemiology for breast cancer is quite different between Taiwanese (ethnically Han Chinese origin) and Caucasian populations [23]. The median age of disease onset is younger in Taiwanese breast cancer patients, and young breast cancer patients in Taiwan carried a greater risk for disease progression and a shorter interval to secondary contralateral breast cancer than in Western women [23–25]. As the early onset and bilaterality of breast cancer are more likely to be related to genetic predisposing factors [26, 27], it is important to identify potential genetic alterations underpinning Taiwanese patients.

The clinical characteristics and outcomes of g*BRCA1/2*-mutant Taiwanese breast cancer patients had been studied in various studies, which revealed a prevalence rate of 3.8% for *BRCA* pathogenic variants in the Taiwanese breast cancer cohort, with a higher proportion among triple-negative breast cancer (TNBC), and an increased risk in contralateral breast cancer [28–31]. However, limited tumor-targeted sequencing analysis had been reported.

Our previous study evaluated mutational profiles in Taiwanese breast cancers by tumor-targeted sequencing. The latest results from the study of 621 enrolled breast cancer patients showed that HR-related genes were altered in 122 (19%) of the population. Other than *BRCA1/2*, the most prevalent HR-related mutant genes were *ARID1A* (7%), *PALB2* (7%), and *PTEN* (6%). In total, 164 (25%) of the 648 Taiwanese breast cancer samples had at least one mutation among the HR-related genes. *BRCA1* and *BRCA2* had affected 3% and 5% of the study population, respectively, and were collectively altered in 6%, with co-occurrence of *BRCA1/2* in 7 breast cancers [32]. In current analysis, we further extended the number of enrolled subjects and evaluated the prevalence of genetic alterations specific in *BRCA1*, *BRCA2*, and *PALB2*, with additional annotation from well-established database.

## Methods

The objective of this study was to assess the prevalence of *BRCA1*, *BRCA2*, and *PALB2* mutations in Taiwanese breast cancer patients using targeted sequencing using tumor-only samples. The Institutional Review Board of Taipei Veterans General Hospital approved the study (protocol number: 2018-09-007A). Written informed consent was obtained from all participants prior to enrollment.

### Study population and patient recruitment

The full protocol of the VGH-TAYLOR study (Veterans General Hospital Taipei—Yung-Ling foundation sinO-canceR study, ClinicalTrials.gov: NCT04626440), which focuses on the heterogeneity of Taiwanese breast cancer patients and included initial targeted sequencing of 380 and 648 assays, has been described elsewhere [32–34].

**Fig. 1:**
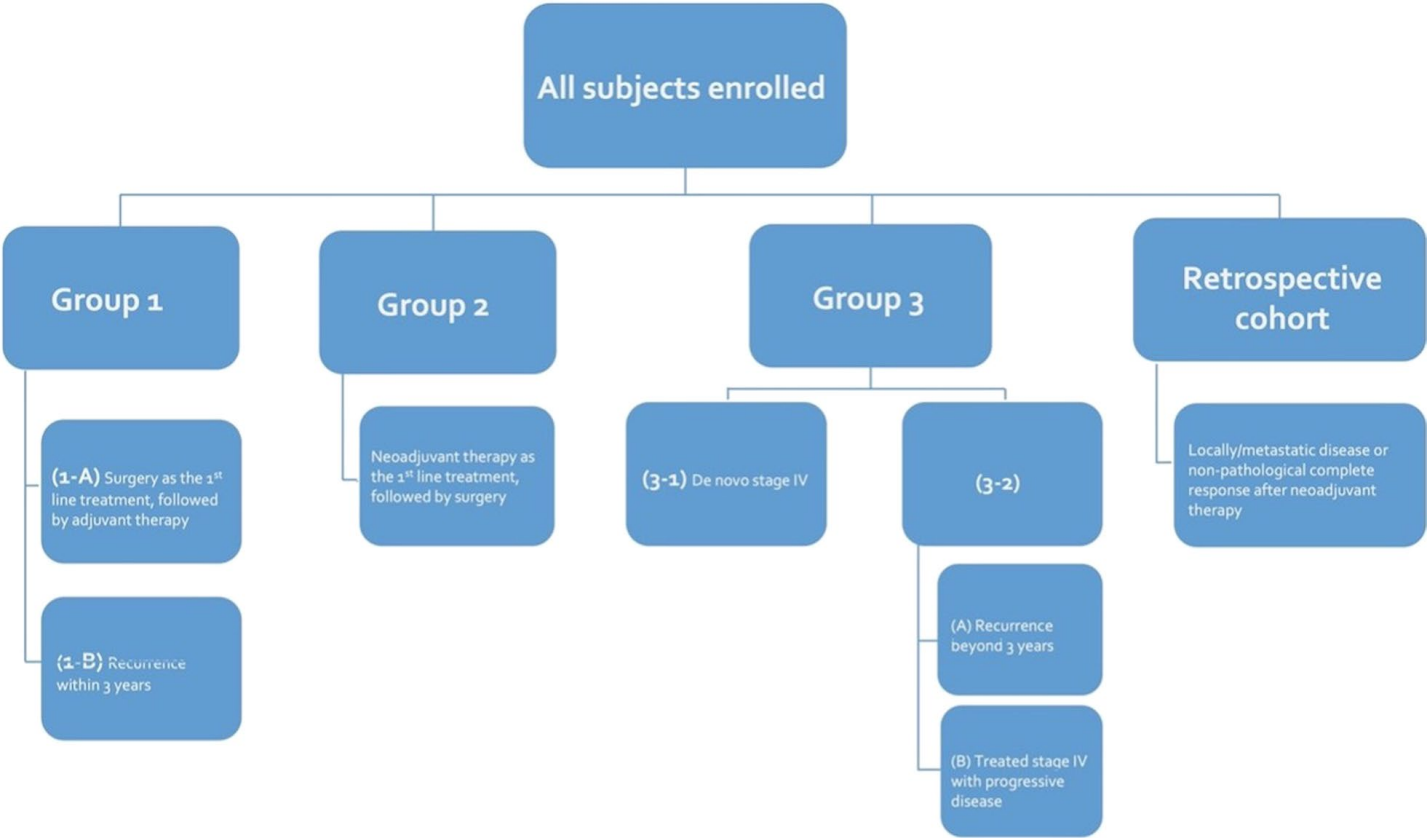
Study protocol of the VGH-TAYLOR study

All patients had been evaluated by their clinicians upon diagnosis. The treatment options were determined by physicians according to patients’ characteristics, molecular subtypes, and clinical stages. The concept of share-decision making (SDM) was fully informed and the process of SDM was carried out before the initiation of treatment. Enrolled subjects were subsequently assigned into Group 1 [planned to receive surgery as the first-line treatment and followed by adjuvant therapy, Group 2 [planned to receive neoadjuvant therapy as the first-line treatment and followed by surgery], and Group 3 [diagnosed with de novo and treatment naïve stage IV breast cancer, or stage IV breast cancer with recurrence beyond three years after surgery] (Fig. 1). Three years of enrollment and 4 years of follow-up after enrollment were planned.

### Collection of breast cancer samples and clinical information

Informed consent was obtained from all participants after a thorough explanation by investigators (CCH and LMT). Clinical parameters were assessed by immunohistochemistry (IHC) with estrogen receptor (ER) and progesterone receptor (PR) positivity defined as at least 1% of tumor cells exhibiting nuclear staining. Hormone receptor positivity was defined as either ER-positive or PR-positive. Patients with human epidermal growth factor receptor II (HER2) testing scored as IHC 3+ (positive) or 2+ (equivocal) and with fluorescence in situ hybridization (FISH)-confirmed amplification were considered HER2-positive. All patients received treatment according to the contemporary practice guidelines of the Comprehensive Breast Health Center at Taipei Veterans General Hospital, which were based on the NCCN and St. Gallen guidelines [35, 36].

### Tumor-only targeted sequencing

The details of tumor only targeted sequencing have been described elsewhere [32, 33]. For next-generation sequencing (NGS) library preparation, the Ion Torre Oncomine™ Comprehensive Assay v3 (Thermo Fisher Scientific, Waltham, MA) was used, enabling the detection of 161 cancer-related genes and identification of single nucleotide variants (SNVs), copy number variations (CNVs), gene fusions, and indels.

A total of 879 consecutive breast cancer patients, representing 924 assays, were enrolled in the study. Formalin-fixed paraffin-embedded specimens were assayed with sequencing data analyzed using the Torrent Suite software. The data were further aligned and annotated by the Ion Reporter with the default Oncomine *BRC*A (5.12) filter applied. Software versions were Torrent Suite (v5.10.0), Ion Reporter (v5.10), Coverage Analysis (v5.10.0.3), SampleID (v5.10.0.1), and VariantCaller (v5.10.0.18). In current study, we focused on *BRCA1*, *BRCA2*, and *PALB2* mutations.

### Variant calling and annotation

Variants were further filtered with the Oncomine™ Knowledgebase Reporter (Thermo Fisher Scientific). To further correct spurious findings due to transethnic discrepancies, the online VariED tool was consulted to filter out Taiwan Biobank polymorphisms [37]. The mutational consequences of filtered variants were determined with the ClinVAR database, a freely accessible public archive of reports on the relationships between human variations and phenotypes [38].

We also used the OncoKB™ to assess the clinical implications of variants that were not identified or confirmed by the Oncomine™ Knowledgebase Reporter or the ClinVAR. The OncoKB™ is a knowledge base for precision oncology that offers information on the therapeutic implications of particular genetic alterations in cancer. The database is curated by a group of oncologists, researchers, and bioinformaticians who meticulously assess the available evidence related to each variant, including clinical trial results, FDA approvals, and guidelines from professional organizations. The OncoKB™ also provides annotations for the functional and structural impact of each variant, as well as the level of evidence supporting the annotation. By using the OncoKB™ to evaluate variants that other databases have not identified or confirmed, we can gain a more thorough understanding of the potential clinical implications of these variants [39]. Tumor sequencing variant annotations were functionally annotated using the SNPnexus [40].

To ensure quality control, over 90% of amplicons with > 100× coverage were used as a parameter. Additionally, a minimum coverage of 250 × was deemed acceptable for detecting SNVs and indels with allele fractions of 10% and 20%, respectively [41, 42]. Actionability was determined based on the joint consensus recommendation of the Association for Molecular Pathology, American Society of Clinical Oncology, and College of American Pathologists [43].

### Reflex germline testing

When patients' tumor samples tested positive for pathogenic or likely pathogenic variants as identified by ClinVar, reflex germline testing was conducted using whole exome sequencing (WES) through blood sample collection. In some cases, whole genome sequencing (WGS) may have been utilized prior to WES. Both WGS and WES are essential in detecting germline mutations linked to a variety of genetic disorders. WGS offers an exhaustive analysis by sequencing the entire genome, allowing for the identification of mutations across both coding and non-coding regions, as well as the detection of structural variations and copy number variations for a detailed examination. In comparison, WES, while more cost-effective and quicker, concentrates on coding regions or exons, potentially overlooking certain mutations that would be detected by WGS.

### Alternative methods for determining germline and somatic mutations

To distinguish germline from somatic mutations with tumor-only sequencing, we employed algorithms including the LOH-germline inference calculator (LOHGIC) [44] and the somatic-germline-zygosity (SGZ) method as alternatives for patients not ready for germline testing [45]. We integrated both methods, considering tumor purity, allele frequency, ploidy, and copy number variations for mutation classification. Only tumor purity and allele frequency were considered as our cohort showed no copy number variations for *BRCA1/2* and all samples were diploid. We simplified both the LOHGIC and the SGZ by directly comparing observed allele frequencies against those expected in germline and somatic mutations.

For germline mutations, expected in both tumor and normal tissues, the allele frequency should be around 50% in pure normal samples and vary in tumor samples due to loss of heterozygosity. In contrast, somatic mutations, present only in tumor cells, will have an allele frequency dependent on tumor purity, typically near 50% in pure tumor samples but lower in samples with less tumor purity intertwined with adjacent normal tissues.

## Result

### Enrolled Taiwanese breast cancers

**Fig. 2:**
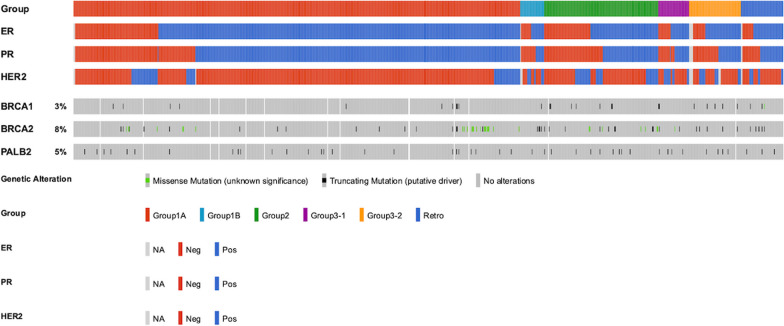
Distributions of clinical variables across study groups

In current study, we presented the updated results of 924 Thermo Fisher (TMO) OCP v3 assays obtained from 879 breast cancer patients, with 43 patients being assayed twice, and 1 patient being assayed thrice from the VGH-TAYLOR study. The patient distributions of clinical scenarios were as follows: Group 1A (surgery first, *n* = 578, 65.8%); Group 1B (recurrence within 3 years, *n* = 22, 2.5%); Group 2 (neoadjuvant therapy, *n* = 117, 13.3%); Group 3–1 (de novo stage IV, *n* = 40, 4.6%); and Group 3–2 (recurrence beyond 3 years, *n* = 67, 7.6%). In addition, there were samples of 55 patients (6.3%) from the retrospective biobank cohort. Figure 2 displays the distributions of IHC results and molecular subtypes.

### Distribution and patterns of *BRCA1, BRCA2, *and *PALB2* mutations

**Fig. 3:**
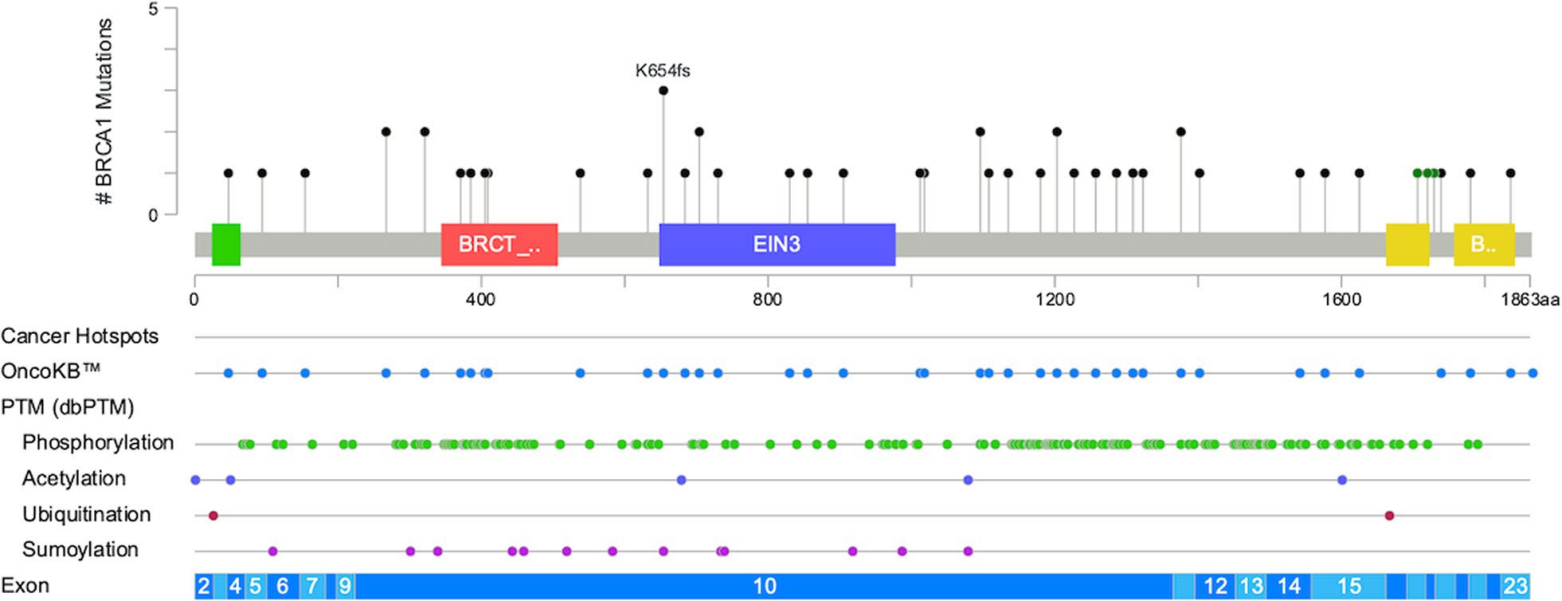
Mutation map of *BRCA1* gene

**Fig. 4:**
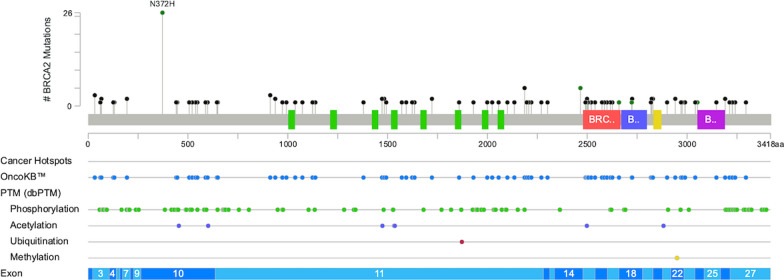
Mutation map of *BRCA2* gene

**Fig. 5:**
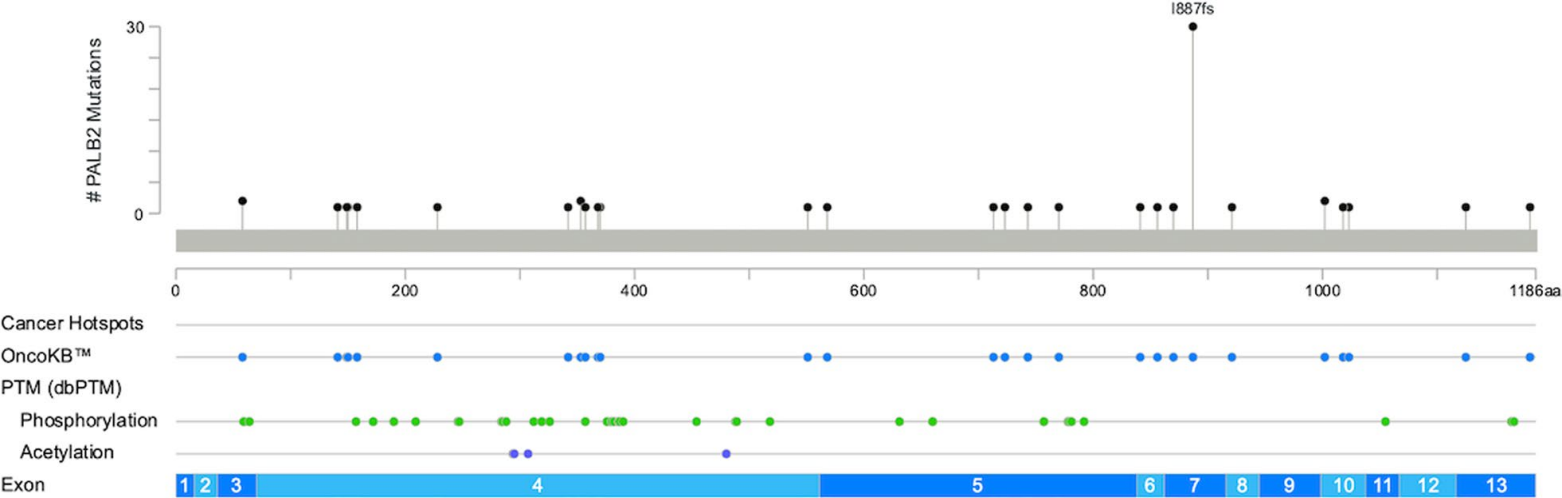
Mutation map of *PLAB2* gene

Of the 924 assays, 281 were positive for mutant *BRCA1*, *BRCA2* and *PLAB2* in 130 patients. These mutations impacted 3.1% (27 patients), 8.6% (76 patients), and 5.2% (46 patients), respectively. In total, genetic alterations were noted in 14.8% (130 patients). Details of distribution and patterns of *BRCA1, BRCA2,* and *PALB2* mutations were portrayed in Table 1, Figs. 3, 4, and 5.

**Table 1:** Characteristic, all, *BRCA1*, *BRCA2*, *PALB2*, stratified by patients. EBC early breast cancer, ABC advanced breast cancer, HR hormone receptor, HER2 human epidermal growth factor receptor 2; TNBC triple-negative breast cancer, OC: ovarian cancer

	Gene status																																							
	Not mutated				Mutated				*p *Value		*BRCA1* (−)				*BRCA1* (+)			*p* Value		*BRCA2* (−)				*BRCA2* (+)				*p* Value		*PALB2* (−)						*PALB2* (+)				*p* Value
	*N*		%		*N*		%				*N*	%			*N*	%				*N*		%		*N*		%				*N*				%		*N*		%		
Total (*N* = 879)	749		85.2%		130		14.8%		–		852	96.9%			27	3.1%		–		803		91.4%		76		8.6%		–		833				94.8%		46		5.2%		–
Group																																								
Retro	48		87.3%		7		12.7%		0.069		53	96.4%			2	3.6%		**0.002**		50		90.9		5		9.1%		0.454		51				92.7%		4		7.3%		0.604
Group 1A	506		87.5%		72		12.5%				569	98.4%			9	1.6%				535		92.6%		43		7.4%				533				95.7%		25		4.3%		
Group 1B	18		81.8%		4		18.2%				21	95.5%			1	4.5%				19		86.4%		3		13.6%				21				95.5%		1		4.5%		
Group 2	91		77.8%		26		22.2%				111	94.9%			6	5.1%				102		87.2%		15		12.8%				109				93.2%		8		6.8%		
Group 3–1	33		82.5%		7		17.5%				38	95.0%			2	5.0%				37		92.5%		3		7.5%				36				90.0%		4		10.0%		
Group 3–2	53		79.1%		14		20.9%				60	89.6%			7	10.4%				60		89.6%		7		10.4%				63				94.0%		4		6.0%		
Stage																																								
EBC	597		85.9%		98		14.1%		0.264		680	97.8%			15	2.2%		**0.002**		637		91.7%		58		8.3%		0.537		662				95.3%		33		4.7%		0.475
ABC	152		82.6%		32		17.4%				172	93.5%			12	6.5%				166		90.2%		18		9.8%				171				92.9%		13		7.1%		
Subtype																																								
HR+/HER2−	482		85.2%		84		14.8%		0.713		553	97.7%			13	2.3%		0.090		515		91.0%		51		9.0%		0.921		536				94.7%		30		5.3%		0.934
HR+/HER2+	78		85.7%		13		14.3%				88	96.7%			3	3.3%				82		90.1%		9		9.9%				87				95.6%		4		4.4%		
HR−/HER2+	67		88.2%		9		11.8%				74	97.4%			2	2.6%				70		92.1%		6		7.9%				73				96.1%		3		3.9%		
TNBC	108		82.4%		23		17.6%				122	93.1%			9	6.9%				122		93.1%		9		6.9%				122				93.1%		9		6.9%		
No data	14		93.3%		1		6.7%				15	100%			0	0%				14		93.3%		1		6.7%				15				100.0%		0		0.0%		
Family history, BC																																								
No BC family history	550		85.8%		91		14.2%		0.719		622	97.0%			19	3.0%		0.920		590		92.0%		51		8.0%		0.204		609				95.5%		32		5.0%		0.891
BC family history (+)	133		83.6%		26		16.4%				154	96.9%			5	3.0%				145		91.2%		14		8.8%				149				93.7%		10		6.3%		
No data	66		83.5%		13		16.5%				76	96.2%			3	3.8%				68		86.1%		11		13.9%				75				94.9%		4		5.1%		
Family history, OC																																								
No OC family history	671		85.9%		110		14.1%		**0.002**		758	97.1%			23	2.9%		0.679		720		92.2%		61		7.8%		**0.011**		741				94.9%		40		5.1%		0.857
OC family history ( +)	8		53.3%		7		46.7%				14	93.3%			1	6.7%				11		73.3%		4		26.7%				13				86.7%		2		13.3%		
No data	70		84.3%		13		15.7%				80	96.4%			3	3.6%				72		86.7%		11		13.3%				79				95.2%		4		4.8%		

Text presented in bold is used to highlight the significance of the *p*-value

The *BRCA1* mutation cohort is associated with a higher proportion of advanced stages compared to those without. Additionally, the *BRCA2*-mutant patients show a higher incidence of family history of ovarian cancer, resulting in a significant difference in the number of mutant patients with a family history of ovarian cancer (Table 1).

In terms of IHC phenotypes, 13 (2.3%) of the *BRCA1* mutant breast cancers were HR+/HER2−, 3 (3.3%) were HR+/HER2+, 2 (2.6%) were HR−/HER2+, and 9 (6.9%) were HR−/HER2−. For *BRCA2* mutated cases, 51 (9.0%) were HR+/HER2−, 9 (9.9%) were HR+/HER2+, 6 (7.9%) were HR−/HER2+, and 9 (6.9%) were HR-/HER2-. Among the *PALB2* mutated patients, 30 (5.3%) were HR+/HER2−, 4 (4.4%) were HR+/HER2+, 3 (3.9%) were HR−/HER2+, and 9 (6.9%) were HR−/HER2− (Table 1).

The study revealed the co-occurrence of *BRCA1/2* in 13 breast cancer samples (log2 odds ratio: > 3, *p*-value < 0.001, and *q*-value < 0.001). Additionally, the co-occurrence of *BRCA1* and *PALB2* was found in 8 samples (log2 odds ratio: > 3, *p*-value < 0.001, and *q*-value < 0.001), and the co-occurrence of *BRCA2* and *PALB2* was found in 8 samples (log2 odds ratio: 2.401, *p*-value < 0.001, and *q*-value < 0.001). These findings are presented in Table 2.

**Table 2:** Mutual exclusivity analysis of *BRCA1*, *BRCA2*, *PALB2* mutations among assayed patients

A	B	Neither	A Not B	B Not A	Both	Log2 Odds Ratio	*p*-Value	*q*-Value	Tendency
*BRCA1*	*BRCA2*	851	15	28	13	> 3	** < 0.001**	** < 0.001**	Co-occurrence
*BRCA1*	*PALB2*	843	20	38	8	> 3	** < 0.001**	** < 0.001**	Co-occurrence
*BRCA2*	*PALB2*	828	33	38	8	2.401	** < 0.001**	** < 0.001**	Co-occurrence

Text presented in bold is used to highlight the significance of the *p*-value

Note: 17 samples were discarded for mutual exclusivity due to missing value in at least one of the interrogated genes

Among these patients, 5 had both *BRCA1/2* mutations, 1 had both *BRCA2* and *PALB2* mutations, none had both *BRCA1* and *PALB2* mutations, and 7 had all three mutations. The list of these patients and the variants that they harbored are shown in Table 3. Twenty-four patients had two or more variants. Of those, 10 patients had dual variants, two had triple variants, one had four variants, three had five variants, one had seven variants, one had eight variants, one had nine variants, one had 15 variants, one had 16 variants, one had 19 variants, one had 24 variants, and one had 25 variants. (Table 3).

**Table 3:** The list of multiple variants per gene or more than one mutant gene

Case no	No. of genes	The list of genes	No. of variants	The list of variants
1	2	*BRCA1* *PALB2*	2	*BRCA1* p.(E1257fs) c.3770_3771delAG *PALB2* p.(I887fs) c.2659_2660delAT
2	1	*BRCA2*	2	*BRCA2* p.(E1571fs) c.4712_4713delAG *BRCA2* p.(N372H) c.1114A > C
3	1	*PALB2*	2	*PALB2* p.(F1181fs) c.3540_3541delAT *PALB2* p.(V870fs) c.2607delC
4	2	*BRCA2* *PALB2*	2	*BRCA2* p.(V2503fs) c.7506_7507insA *PALB2* p.(I887fs) c.2659_2660delAT
5	2	*BRCA1* *PALB2*	2	*BRCA1* p.(K654fs) c.1960_1961insG *PALB2* p.(M723fs) c.2167_2168delAT
6	1	*PALB2*	2	*PALB2* p.(P713fs) c.2138delC *PALB2* p.(Y743*) c.2229T > A
7	1	*BRCA2*	2	BRCA2 p.(Q2499*) c.7495C > *T* *BRCA2* c.476-3C > T
8	2	*BRCA1* *BRCA2*	2	*BRCA1* p.(R1720Q) c.5159G > A *BRCA2* p.(W2970*) c.8910G > A
9	1	*BRCA2*	2	*BRCA2* p.(S2186fs) c.6556_6557insA *BRCA2* p.(X159_splice) c.476-2A > G
10	2	*BRCA1* *BRCA2*	2	*BRCA1* p.(S267fs) c.799_800insT *BRCA2* p.(T912fs) c.2734_2735insA
11	1	*BRCA2*	3	*BRCA2* p.(L1635*) c.4904 T > A *BRCA2* p.(S2186fs) c.6556_6557insA *BRCA2* p.(X159_splice) c.476-2A > G
12	2	*BRCA1* *BRCA2*	3	*BRCA1 c.5137 + 1G > A *BRCA2* p.(Q2539*) c.7615C > T *BRCA2* p.(X2659_splice) c.7976 + 2C > T
13	2	*BRCA2* *PALB2*	4	*BRCA2* p.(E1493fs) c.4477delG *BRCA2* p.(Q3227*) c.9679C > T *BRCA2* p.(W194*) c.582G > A *PALB2* p.(E1018*) c.3052G > T
14	2	*BRCA1* *BRCA2*	5	*BRCA1* p.(T1376fs) c.4126_4127insA *BRCA2* p.(E33*) c.96_97insT *BRCA2* p.(S3041fs) c.9121_9122insT *BRCA2* p.(S3147fs) c.9439_9440insT *BRCA2* p.(T912fs) c.2734_2735insA
15	2	*BRCA1* *BRCA2*	5	*BRCA1* p.(Q855*) c.2563C > T *BRCA1* p.(X1760_splice) c.5341-3C > T *BRCA2* p.(Q1124*) c.3370C > T *BRCA2* p.(Q126*) c.376C > T *BRCA2* p.(Q3295*) c.9883C > T
16	3	*BRCA1* *BRCA2* *PALB2*	5	*BRCA1* p.(Q1867*) c.5599C > T *BRCA2* p.(Q2829*) c.8485C > T *BRCA2* p.(Q649*) c.1945C > T *BRCA2* c.6842-1G > A *PALB2* c.2586 + 1G > A
17	3	*BRCA1* *BRCA2* *PALB2*	7	*BRCA1* p.(X27_splice) c.80 + 1G > A *BRCA1* p.(Q1577*) c.4729C > T *BRCA2* p.(R3052Q) c.9155G > A *BRCA2* p.(R2520*) c.7558C > T *PALB2* p.(Q141*) c.421C > T *PALB2* p.(Q228*) c.682C > T *PALB2* p.(Q921*) c.2761C > T
18	3	*BRCA1* *BRCA2* *PALB2*	8	*BRCA1* p.(S1180fs) c.3538_3539insA *BRCA2* p.(S538fs) c.1612_1613insA *BRCA2* p.(S973fs) c.2916_2917insA *BRCA2* p.(T598fs) c.1792_1793insA *PALB2* p.(D1125fs) c.3372_3373insA *PALB2* p.(N342fs) c.1025_1026insA *PALB2* p.(N368fs) c.1103_1104insA *PALB2* p.(S357fs) c.1068_1069insA
19	2	*BRCA1* *BRCA2*	10	*BRCA1* p.(Q1227*) c.3679C > T *BRCA1* p.(Q1625*) c.4873C > T *BRCA1* p.(Q1867*) c.5599C > T *BRCA1* p.(W1739*) c.5216G > A *BRCA1* p.(W1739*) c.5217G > A *BRCA2* p.(X2985_splice) c.8953 + 1G > A *BRCA2* p.(D2723N) c.8167G > A *BRCA2* p.(Q66*) c.196C > T *BRCA2* p.(W3191*) c.9572G > A *BRCA2* p.(W993*) c.2978G > A
20	2	*BRCA1* *BRCA2*	15	*BRCA1* p.(X1366_splice) c.4096 + 1G > A *BRCA1* p.(X1559_splice) c.4738 + 1G > A *BRCA1* p.(X183_splice) c.547 + 1G > A *BRCA1* p.(W321*) c.963G > A *BRCA2* p.(X106_splice) c.317-3C > T *BRCA2* p.(G2313D) c.6938G > A *BRCA2* p.(X2602_splice) c.7806-3C > T *BRCA2* p.(E2220fs) c.6658delG *BRCA2* p.(Q2100*) c.6298C > T *BRCA2* p.(Q2491*) c.7471C > T *BRCA2* p.(Q2506*) c.7516C > T *BRCA2* p.(Q2823*) c.8467C > T *BRCA2* p.(Q66*) c.196C > T *BRCA2* p.(R2494*) c.7480C > T *BRCA2* p.(W2990*) c.8969G > A
21	3	*BRCA1* *BRCA2* *PALB2*	16	*BRCA1* p.(Q1135*) c.3403C > T *BRCA1* p.(Q538*) c.1612C > T *BRCA1* p.(Q905*) c.2713C > T *BRCA1* p.(T1706I) c.5117C > T *BRCA1* p.(W385*) c.1155G > A *BRCA2* p.(X2602_splice) c.7805 + 1G > A *BRCA2* p.(Q1379*) c.4135C > T *BRCA2* p.(Q1623*) c.4867C > T *BRCA2* p.(Q2823*) c.8467C > T *BRCA2* p.(R2659K) c.7976G > A *BRCA2* p.(W194*) c.581G > A *BRCA2* p.(W2574*) c.7722G > A *BRCA2* p.(W2586*) c.7758G > A *BRCA2* p.(W2725*) c.8174G > A *PALB2* p.(Q1023*) c.3067C > T *PALB2* p.(Q856*) c.2566C > T
22	3	*BRCA1* *BRCA2* *PALB2*	19	*BRCA1* p.(X71_splice) c.212 + 1G > A *BRCA1* c.4096 + 1G > A c.4096 + 1G > A *&*BRCA1* c.5215 + 1G > A *&*BRCA1* c.5256 + 1G > A *BRCA1* p.(X183_splice) c.547 + 1G > A *BRCA1* p.(A1729V) c.5186C > T *BRCA1* p.(W1836*) c.5507G > A *BRCA2* p.(X142_splice) c.425 + 1G > A *BRCA2* c.8488-1G > A c.8488-1G > A *BRCA2* p.(X3217_splice) c.9649-3C > T *BRCA2* p.(Q1138*) c.3412C > T *BRCA2* p.(Q2024*) c.6070C > T *BRCA2* p.(W2626*) c.7878G > A *PALB2* p.(X839_splice) c.2515-3C > T *PALB2* p.(X1038_splice) c.3114-1G > A *PALB2* p.(X1117_splice) c.3351-3C > T *PALB2* p.(E1002fs) c.3004delG *PALB2* p.(Q370*) c.1108C > T *PALB2* p.(Q568*) c.1702C > T
23	3	*BRCA1* *BRCA2* *PALB2*	24	*BRCA1* p.(K1780fs) c.5339_5340insA *BRCA1* p.(N1542fs) c.4625_4626insA *BRCA1* p.(Q1096fs) c.3285_3286insA *BRCA1* p.(Q94fs) c.279_280insT *BRCA1* p.(R1012fs) c.3034_3035insA *BRCA1* p.(S267fs) c.799_800insT *BRCA1* p.(T1376fs) c.4126_4127insA *BRCA1* p.(V409fs) c.1224_1225insA *BRCA2* p.(E33*) c.96_97insT *BRCA2* p.(L446fs) c.1337_1338insT *BRCA2* p.(Q2499fs) c.7494_7495insA *BRCA2* p.(Q2941fs) c.8820_8821insA *BRCA2* p.(Q937fs) c.2808_2809insA *BRCA2* p.(R645fs) c.1933_1934insA *BRCA2* p.(S2056fs) c.6164_6165insT *BRCA2* p.(S2976fs) c.8926_8927insA *BRCA2* p.(T1483fs) c.4447_4448insA *BRCA2* p.(T1858fs) c.5572_5573insA *BRCA2* p.(T2197fs) c.6589_6590insA %*BRCA2* p.(T441fs) c.1317delT, c.1320_1321insT *BRCA2* p.(T912fs) c.2734_2735insA *PALB2* p.(E1002fs) c.3003_3004insA *PALB2* p.(L58fs) c.173_174insT *PALB2* p.(T841fs) c.2521_2522insA
24	3	*BRCA1* *BRCA2* *PALB2*	25	*BRCA1* p.(E730fs) c.2187_2188insA *BRCA1* p.(H1402fs) c.4203_4204insA *BRCA1* p.(I1108fs) c.3322_3323insA *BRCA1* p.(N1018fs) c.3053_3054insA *BRCA1* p.(N1309fs) c.3926_3927insA *BRCA1* p.(P371fs) c.1110_1111insT *BRCA1* p.(P684fs) c.2048_2049insA *BRCA1* p.(Q1096fs) c.3285_3286insA *BRCA1* p.(Q1323fs) c.3966_3967insA *BRCA2* p.(D2819*) c.8454_8455insT *BRCA2* p.(D2900fs) c.8697_8698insA *BRCA2* p.(E1593fs) c.4776_4777insA *BRCA2* p.(E2301fs) c.6900_6901insA *BRCA2* p.(E33*) c.96_97insT *BRCA2* p.(L61fs) c.181_182insA *BRCA2* p.(N3213fs) c.9638_9639insA *BRCA2* p.(Q2941fs) c.8820_8821insA *BRCA2* p.(Q937fs) c.2808_2809insA *BRCA2* p.(S131fs) c.391_392insT *BRCA2* p.(S2616fs) c.7846_7847insT *BRCA2* p.(S3241fs) c.9721_9722insT *BRCA2* p.(T1483fs) c.4447_4448insA *BRCA2* p.(T2207fs) c.6619_6620insA *PALB2* p.(A770fs) c.2307_2308insT *PALB2* p.(L58fs) c.173_174insT

^*^No amino acid (AA) change was found

^&^The novel variants

%Two different codings resulted into the same amino acid (AA) change

**Fig. 6:**
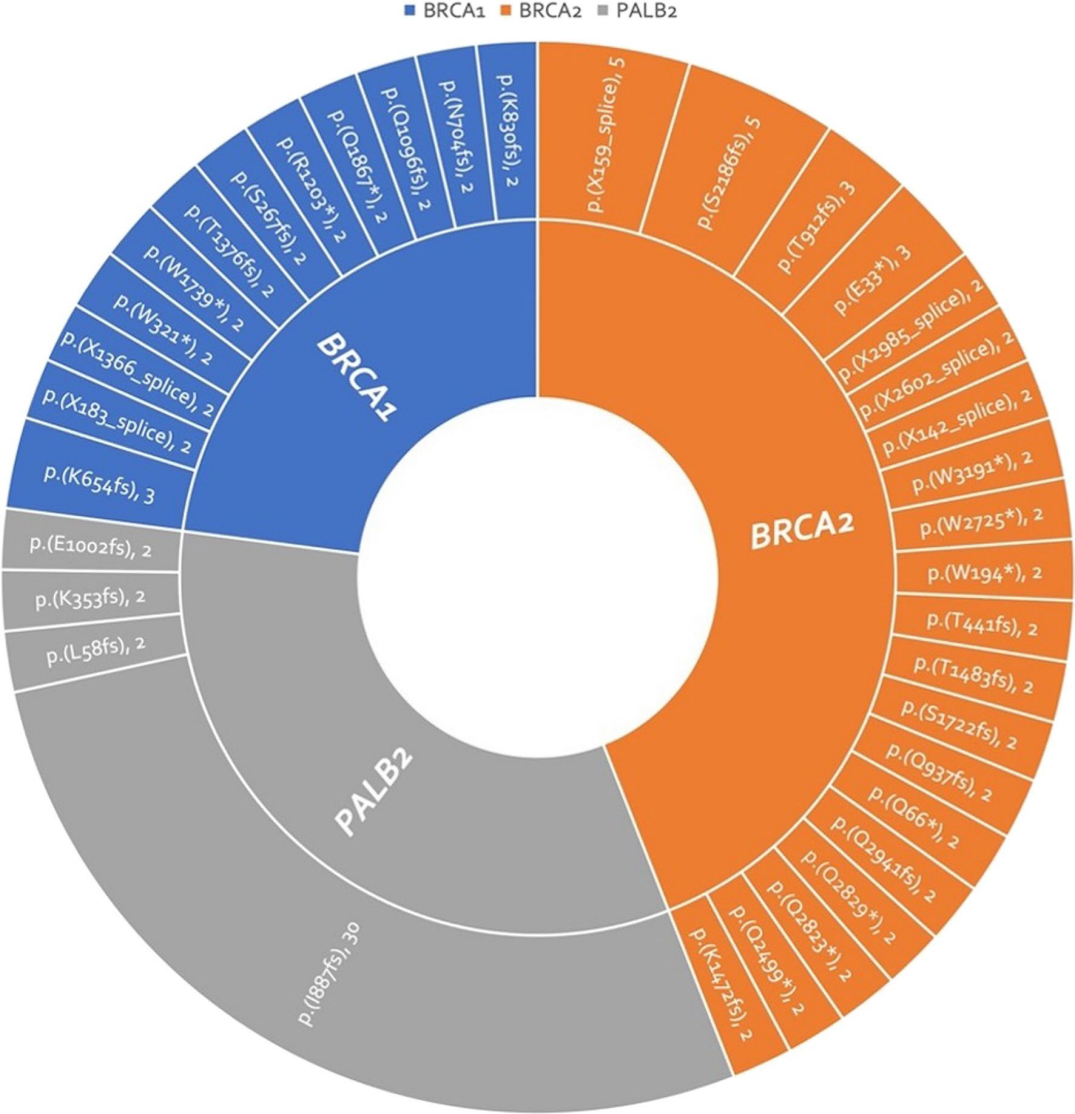
Amino acid changes, repeatedly mutated, with evidence of clinical implication, validated by the OncoKB™

Among all the variants, p.I887fs was observed 30 times in *PALB2*-mutant assays. For *BRCA2*-mutant assays, p.S2186fs, p.V2466A, and p.X159_splice were observed 5 times, while p.T912fs and p.E33* were observed 3 times each. In *BRCA1*-mutated assays, only p.K654fs was observed three times, while other variants were observed no more than twice. It should be noted that although p.N372H was observed 26 times in *BRCA2*-mutated assays, it has been confirmed to be a benign variant. The list of those recurrent variants with clinical implications is displayed in Fig. 6.

### Functional annotations and clinical implications

The study analyzed various genetic variants and identified 176 amino acid (AA) changes, and the characteristics stratified by genes and the clinical implications are listed in Table 2. There were four variant that did not notice any AA change, and three novel splice site variants (*BRCA1* c.5256+1G > A, *BRCA1* c.5215+1G > A, and *BRCA2* c.-38-3CAG > C) were identified, all listed in Table 4. Although these novel variants had no AA change, they were classified as Pathogenic/Likely pathogenic (P/LP) by the ClinVAR database. Notably, 60.2% (106) of the discovered AA changes were not documented in either ClinVAR or the Oncomine™ Knowledge database. Using the OncoKB™ for annotation, 171 (97.2%) AA changes were found to have clinical implications. Half of all missense mutations without clinical implications (4 out of 8) were deemed insignificant, and 23.1% (3 out of 13) of AA changes not recorded in the ClinVAR or the Oncomine™ Knowledge database was not considered clinically relevant. The *BRCA2* mutation cohort exhibited the highest proportion of P/LP variants. This cohort also contained the only benign variant identified and had the highest number of AA changes without clinical implications, as per the OncoKB™ (Table 5).

**Table 4:** The list of novel variants discovered in the cohort

	Gene	Site	Location	Transcript	Coding	Allele Frequency	ClinVAR	Oncomine	OncoKB™
1	*BRCA1*	splicesite	chr17:41215349	NM_007300.3	c.5256+1G > A	12.53%	Pathogenic	Not recorded	No reference
2	*BRCA1*	splicesite	chr17:41215890	NM_007300.3	c.5215+1G > A	6.12%	Pathogenic	Hotspot	No reference
3	*BRCA2*	splicesite	chr13:32890556	NM_000059.3	c.-38-3CAG > C	51.19%	Likely pathogenic	Not recorded	No reference

**Table 5:** Summary of amino acid changes in study cohort, stratified by evidence of clinical implication

	No clinical implication		With clinical implication		Sum	*p* Value
	*N*	%	*N*	%	*N*	
Total	5	2.8%	171	97.2%	176	–
Gene						
* BRCA1*	1	2.0%	48	98.0%	49 (27.8%)	0.415
* BRCA2*	4	4.3%	90	95.7%	94 (53.4%)	
* PALB2*	0	0.0%	33	100.0%	33 (18.8%)	
Site						
Exonic	4	2.7%	146	97.3%	150 (85.2%)	0.738
Splicesite	1	3.8%	25	96.2%	26 (14.8%)	
Type of mutation						
Frameshift deletion	0	0.0%	28	100.0%	28 (15.9%)	** < 0.001**
Frameshift insertion	0	0.0%	52	100.0%	52 (29.5%)	
Missense	4	50.0%	4	50.0%	8 (4.5%)	
Nonsense	0	0.0%	62	100.0%	62 (35.2%)	
Unknown	1	3.8%	25	96.2%	26 (14.8%)	
Annotation, by Clinvar						
Pathogenic	0	0.0%	68	100.0%	68 (38.6%)	** < 0.001**
Likely Pathogenic	0	0.0%	5	100.0%	5 (2.8%)	
Pathogenic/Likely Pathogenic	0	0.0%	6	100.0%	6 (3.4%)	
Conflicting	2	50.0%	2	50.0%	4 (2.3%)	
Benign	1	100.0%	0	0.0%	1 (0.6%)	
Uncertain significance	1	33.3%	2	66.7%	3 (1.7%)	
Unknown	1	1.1%	88	98.9%	89 (50.6%)	
Annotation, by Oncomine						
No annotation	3	13.6%	19	86.4%	22 (12.5%)	** < 0.001**
Deleterious	0	0.0%	142	100.0%	142 (80.7%)	
Hotspot	2	16.7%	10	83.3%	12 (6.8%)	
Annotation, comparing ClinVar to Oncomine						
ClinVar(+), Oncomine(+)	0	0.0%	70	100.0%	70 (39.8%)	** < 0.001**
ClinVar(+), Oncomine(−)	0	0.0%	9	100.0%	9 (5.1%)	
ClinVar(−), Oncomine(+)	2	2.4%	82	97.6%	84 (47.7%)	
ClinVar(−), Oncomine(−)	3	23.1%	10	76.9%	13 (7.4%)	

Text presented in bold is used to highlight the significance of the *p*-value

### Reflex germline testing

In our study, reflex germline testing was conducted for patients until November 20, 2023. These procedures are summarized in Table 6, which concentrates on WGS and WES analyses for individuals who had positive results from tumor-only sequencing. Specifically, 48 cases, constituting 36.9%, were identified with pathogenic or likely pathogenic variants via tumor-targeted sequencing, as classified by the ClinVar database, and were subsequently recalled for further investigation through WGS or WES.Among 130 cases examined, 7 cases (5.4%) completed WGS uncovering crucial genetic variations. None harbored germline mutations in *BRCA1/2* and *PALB2*. Another 20 cases (15.4%) were reached and 9 had completed WES (6.9%). The study group encountered enormous challenges including loss of follow-up and 14 were deceased.

**Table 6:** Characteristic of the cases undergoing reflex germline testing

Items	*N* (cases)	%
Total cases	130	100
Cases that underwent whole genome sequencing (WGS)	7	5.4
Cases with pathogenic or likely pathogenic variants detected through tumor-only sequencing, planned for whole exome sequencing (WES)	48	36.9
Possible candidates for whole exome sequencing (WES)	20	15.4
Cases that underwent whole exome sequencing (WES)	9	6.9
Cases loss of follow up	5	3.8
Cases with no further clinical arrangements	9	6.9
Cases expired	14	10.8
Germline mutation detected, pathogenic variant	4	3.1
Germline mutation detected, uncertain significance	2	1.5
Germline mutation detected, benign	5	3.8
Pending result of whole genome sequencing (WGS) or whole exome sequencing (WES)	5	3.8

The combined results of whole genome sequencing (WGS) and whole exome sequencing (WES) have provided in-depth insights into the origins of identified variants. Notably, 4 cases (3.1%) exhibited pathogenic germline mutations. Reflex germline testing results showed that three variants (*BRCA1* c.1969_1970del, *BRCA1* c.3629_3630del, *BRCA2* c.8755-1G > C) were classified as Pathogenic/Likely pathogenic (P/LP) by ClinVar and as likely loss-of-function or likely oncogenic by OncoKB. Meanwhile, one variant (*PALB2* c.448C > T) was not listed in ClinVar, but OncoKB annotated it as likely loss-of-function or likely oncogenic. Additionally, there were 2 cases (1.5%) of germline mutations with uncertain significance, and 5 cases (3.8%) with benign germline alterations, which emphasize the genetic intricacy involved in the development of breast cancer. The result for all the variants from reflex germline testing is presented in Additional file 1: Table S1.

### Alternative methods for determining germline and somatic mutations

Table 7 illustrates the results of LOHGIC and SGZ analyses. Among the 281 samples, 40 were identified as germline mutations and 169 samples (60.1%) were of somatic origin. Borderline cases comprised 26 samples (9.3%). Lastly, there were 46 samples, (16.4%) unclassifiable due to missing data in tumor purity.

**Table 7:** The results of the prediction of germline/somatic mutations by the simplified LOHGIC and SGZ method

Categories	Total samples	%	*BRCA1*	*BRCA2*	*PALB2*
Germline	40	14.2	8	32	0
Somatic	169	60.1	50	96	22
Borderline	26	9.3	4	20	2
No data	46	16.4	3	2	41

## Discussion

Our study presents the one of the largest cohorts of breast cancer patients with *BRCA1*, *BRCA2*, and *PALB2* mutations detected through tumor-only sequencing in Taiwan. We analyzed 924 Thermo Fisher OCP v3 assays from 879 breast cancer patients, dividing them into different groups based on their clinical scenarios. Out of the 924 assays conducted, 281 were positive for mutant genes in 130 patients, with *BRCA1*, *BRCA2*, and *PALB2* mutations identified in 27 patients (3.1%), 76 patients (8.6%), and 46 patients (5.2%), respectively. Overall, genetic alterations were observed in 14.8% of the assays. The high detection rate of breast cancer susceptibility genes could lead to more patients undergoing germline testing and receiving appropriate treatments.

### Genetic mutations and variants

Recent research has revealed a significant occurrence of harmful variants in three key genes associated with breast cancer risk, as analyzed through tumor genomic profiling. In a significant study by the Breast Cancer Association Consortium in 2021 involving over 60,000 breast cancer cases and 53,000 controls, sequencing was conducted on 34 potential risk genes. The standout genes were *BRCA1*, *BRCA2*, and *PALB2*. Variants leading to truncating/incomplete proteins in these genes correlated with an increased breast cancer risk, which were statistically significant (all *P*-values less than 0.0001), underscoring their crucial role in the genetic landscape of breast cancer. These genes are also recognized markers for determining the eligibility for PARP inhibitor therapies, warranting further investigation into their contribution to breast cancer risk [46]. The frequency of mutations in these genes varies by the method of testing and the population studied. Research focusing on Asian patients with *BRCA1/2* mutations found a wide prevalence range, with *BRCA1* mutations present in 2.3–42% and *BRCA2* mutations in 2.3–11.4% of the group [47]. In Taiwan, a study showed a 3.8% prevalence of these mutations in an unselected patient population [30]. Reports on *PALB2* mutations in Asian populations are scarce; however, a thorough global review indicated that *PALB2* pathogenic variants occur in 0.9% to 3.2% [19].

While germline mutations in breast cancer susceptibility genes have been extensively studied, reports of somatic mutations are rare. However, with the advent of tumor-targeted sequencing, we can explore both germline and somatic mutations simultaneously. In 2022, the Dana-Farber/Harvard Cancer Center conducted a tumor-targeted sequencing study across a broad range of malignancies on 7575 patients, which included 1514 breast cancer patients. If a patient was identified with any P/LP variants of *BRCA1*, *BRCA2*, or *PALB2* within the tumor, clinical germline testing (CGT) would be performed. The study found that *BRCA1* and *BRCA2* mutations were present in 2.5% and 3.7% of breast cancer patients, respectively, while *PALB2* mutations were present in 0.6% of then. Out of all P/LP variants from tumor-sequencing, 70.5% were confirmed as germline mutations [48].

The study analyzed various genetic variants and identified 176 amino acid (AA) changes. All *BRCA1*, *BRCA2*, and *PLAB2* mutation information was curated and confirmed by the ClinVAR, the Oncomine™ Knowledgebase Reporter, and the OncoKB™. There were 4 variants that did not have AA changes reported, and 3 of them were novel variants (*BRCA1* c.5256+1G > A, *BRCA1* c.5215+1G > A, and *BRCA2* c.-38-3CAG > C), which were reported the first time in this Taiwanese cohort. The most common *BRCA1* mutation was p.K654fs (c.1960_1961insG), which is a frameshift insertion and deleterious mutation (three cases). Although not recorded by the ClinVAR or the ACMG 73 genes the OncoKB™ has confirmed its clinical implication. The most common *BRCA2* mutations were p.N372H (c.1114A > C, 26 cases), p.S2186fs (c.6556_6557insA; 5 cases), p.V2466A (c.7397T > C; 5 cases), and p.X159_splice (c.476−2A > G/c or 476−3C > T; 5 cases). The variant p.N372H was initially reported in 2000 and is one of the common non-synonymous polymorphisms [49, 50]. It has been a research focus in the scientific community and has drawn increasing attention [50–58]. A meta-analysis of 22 studies, involving 22,515 cases and 22,388 controls, found no significant association between the *BRCA2* p.N372H polymorphism and breast cancer risk. This suggests that the *BRCA2* p.N372H allele may be non-pathogenic. Although p.S2186fs is not annotated by the ClinVAR or listed in the ACMG 73 genes, it is a frameshift insertion and deleterious mutation with clinical implications. This has been confirmed by the OncoKB™. For p.V2466A, the ClinVAR provides conflicting interpretations, with some considering it a benign entity. Regarding p.X159_splice, it is interesting to note that there are two different coding variants being recorded. c.476-3C > T has conflicting implications and has been seen in patients from Central/Eastern Europe. In contrast, c.476-2A > G has been ascertained as pathogenic and has been mentioned in Italian and Chinese population. The most common *PALB2* mutation observed was p.I887fs (c.2659_2660delAT), a deleterious frameshift mutation that was observed in 30 patients. Although this mutation is not listed in the ClinVAR or ACMG 73 genes list, its clinical implications have been approved by the OncoKB™.

### Gene–gene interactions

The study revealed a significant tendency of co-occurrence between *BRCA1/2*, *BRCA1-PALB2*, and *BRCA2-PALB2* mutations. It should be noted that 17 samples were discarded due to missing values in at least one of the interrogated genes, preventing analysis of mutual exclusivity. Constructing a gene–gene interaction (GGI) network is important for understanding breast carcinogenesis, as single gene or protein alterations are not sufficient to induce cancer. Rather, the interactions with other genes or microenvironment play a key role. A large-scale study on the interaction between genes was conducted on European Non-Small Cell Lung Cancer (NSCLC) risk, using a total of 445,221 participants from various projects [59]. The study found important gene–gene interactions in the 5p15.33 and 6p21.32 regions, which can be used to improve lung cancer screening models.

It was found that *BRCA1* interacts with *RAD51* to play a role in DNA repair, while *BRCA2* co-localizes with *RAD51* and *BRCA1*, indicating a similar function [60]. *PALB2*, first reported by Xia et al. in 2006 [61], plays a fundamental role in HR. It acts as a bridging molecule that connects the *BRCA* complex (*BRCA1-PALB2-BRCA2-RAD5*1*)* and facilitates the function of *RAD51*, a protein that is vital for strand invasion during HR [18, 19].

A large-scale mutational analysis in 7,325 individuals identified four interactions between mutations in the breast cancer susceptibility genes [62]. These interactions include *ATM* and *CHEK2* with *BRCA1* and *BRCA2*, *ATM* and *BRCA*, *CHEK2* and *BRCA1*/*BRCA2* combined, and *CHEK2* and *BRCA1* or *BRCA2*. The results show a lower risk of breast cancer than that predicted by the multiplicative product of the constituent risks. These findings likely reflect functional relationships between the encoded proteins in DNA repair and have important implications for models of disease predisposition and clinical translation.

Currently, limited studies have addressed the gene–gene interaction among *BRCA1*, *BRCA*, and *PALB2* in real-world settings of large-scale breast cancer gene analysis. Our results may shed light on exploring the association between breast cancer susceptibility genes and the possibility of creating a genetic panel for predicting and prognosing hereditary breast cancer.

### Clinical features

The *BRCA1* mutation cohort has a higher proportion of advanced-stage disease compared to others, which may be due to various reasons. One possible explanation is that patients with *BRCA1* mutations are more likely to have triple-negative breast cancer [63], which is associated with a higher risk of distant recurrence [64, 65]. Another possible reason is that *BRCA1* mutations may be associated with a higher likelihood of developing bilateral breast cancer, which could increase the risk of disease progression [66]. Additionally, patients with *BRCA1* mutations are more likely to develop breast cancer at a younger age, when the disease may be more aggressive and therefore more likely to be diagnosed at an advanced stage [67, 68]. Despite having more advanced disease, evidence is mixed as to whether *BRCA*-associated breast cancer has poorer outcomes. Some studies have shown that carriers of a *BRCA1* mutation have worse overall survival [69], while others have found no significant difference in outcomes [70].

### Clinical implications and practice of tumor-targeted sequencing

Nowadays, tumor-only targeted sequencing has gained increasing attention. One advantage of tumor-only testing is that it can reveal P/LP variants in genes associated with cancer predisposition and potential therapeutics with a higher level of coverage. Identification of mutations through tumor-only sequencing may lead to reflex germline testing, which can identify individuals at an increased risk for cancer and allow for early detection and intervention.

Recent studies have indicated that direct tumor sequencing could be more advantageous than solely testing for inherited mutations. The comprehensive tumor sequencing effort followed by clinical genetic testing at Dana-Farber/Harvard Cancer Center has not only highlighted the commonness of pathogenic/likely pathogenic mutations in genes such as *BRCA1*, *BRCA2*, and *PALB2*, but has also underscored the significance of this targeted sequencing approach [48]. They found that patients with *BRCA* mutations were more likely to undergo CGT than those without. Interestingly, over half (52.9%) of the tumor-identified P/LP patients did not meet any personal or family history criteria for CGT. Additionally, 32.7% of patients with *BRCA1/2* or *PALB2* P/LP variants did not have any other clinical indication for germline testing. Nonetheless, 70.5% of P/LP variants identified through CGT were germline origin. These results show the potentiality of tumor-only sequencing in detecting P/LP mutations in cancer predisposition genes across malignancies. Furthermore, they highlighted the necessity of expanding the indications for CGT beyond traditional criteria. A significant proportion of patients with these mutations may not have any personal or family history of cancer, which may have a significant impact on cancer risk assessment, surveillance, and treatment decisions in the future. Before universal germline and tumor sequencing becomes feasibility, tumor-targeted sequencing seems to be a reasonable choice for personalized therapy.

Both germline and somatic alterations can affect treatment decisions and outcomes. For breast cancer patients, the results of the TBCRC-048 and TBB trials suggested further exploration of PARP inhibitors in metastatic or advanced breast cancers with HR-associated mutations beyond *BRCA1* and *BRCA2* [16, 17]. Identifying additional biomarkers to expand this treatment in somatic *BRCA1/2*-mutant or HR-related-gene-mutant advanced breast or ovarian cancers could significantly benefit patients who would otherwise receive chemotherapies as the only regimen. These efforts may reveal a patient population that would benefit from targeted therapy, improving patient outcomes and reducing the complications associated with cytotoxic chemotherapy.

### Reflex germline testing and other alternative methods for identifying the origin of variants

The significance and clinical applicability of tumor-targeted sequencing is well acknowledged, yet the unique importance of reflex germline testing cannot be overemphasized. We are continuously expanding cases for such testing. However, the extent of reflex testing conducted to date is still limited, with only 7 cases underwent WGS and 9 with WES (Table 6). This has led us to explore alternative methods to determine whether the reported mutation is germline or somatic origin.

The refined LOHGIC and SGZ methodologies are designed to assess three crucial factors: tumor purity (the proportion of cancer cells in a sample), allele frequency (the incidence of mutations in DNA sequencing reads), and confirmation of a diploid genome (maintaining two copies of each gene). These methods are crucial for differentiating between somatic mutations, which occur in somatic (non-reproductive) cells, and germline mutations, which are inherited. Although these algorithms are useful, they have their limitations; in an analysis of 130 cases, 10 could not be conclusively classified as either germline or somatic due to varying origins of the mutations. Nonetheless, there was a noticeable pattern, with 60% of the mutations being identified as somatic. This observation highlights the imperative for more refined techniques to accurately determine the origins of mutations.

### Annotation and curation

In the study, 60.2% (106) of the reported AA changes were not documented in either the ClinVAR or the commercial the Oncomine™ Knowledge base Reporter. However, when using the OncoKB™ for annotation, 171 AA changes were found to have clinical implications. Nearly half (*N* = 80, 45.5%) of these variants with AA changes were due to frameshift deletions or insertions, which were all clinically significant according to the OncoKB™. Interestingly, half of missense mutations without clinical implications (4 out of 8) were deemed insignificant, and 23.1% (3 out of 13) of AA changes not recorded in the ClinVAR or the Oncomine™ Knowledge base Reporter were not considered clinically relevant. Regarding the 40 splice site mutations, 62.5% (25) were not reported as deleterious mutations by the Oncomine™ Knowledgebase Reporter, but the ClinVAR identified 60% (15 out of 25) of these mutations as P/LP variants. Furthermore, the OncoKB™ identified that 85% (34 out of 40) of all splice site mutations to be clinically significant.

Accurate interpretation of genetic variants is critical in both clinical and research settings. Before reporting detected variants, appropriately trained and certified molecular diagnostic procedures must be carefully carried out in the context of clinical scenarios, including histologic features [43]. However, the classification criteria can vary between submitters, and the evidence for a particular variant may be conflicting, leading to difficulties in an unbiased interpretation. Numerous studies have explored potential indicators for reinterpreting pathogenic variants within specific databases, as well as across distinct platforms. For example, in a recent comparison of the RefSeq and Gencode human gene databases, only 27.5% of transcripts annotated in the Gencode are shared by the RefSeq [71]. Whiffin et al. selected 43 variants from the ClinVAR classified as P/LP, which were not rare enough in at least one of the Exome Aggregation Consortium populations [72]. Their analysis showed that 42 of these variants should be considered variants of uncertain significance (VUS) instead of P/LP. Xiang et al. analyzed common P/LP variants in the ClinVAR database and identified indicators associated with reclassification, indicating missing opportunities due to misinterpretation [73]. The study selected 217 variants in 173 genes for manual interpretation according to guidelines, with 40% of which downgraded to benign or VUS, while 2% identified as more likely risk alleles. Inappropriate classification was associated with low-rank, older annotation, higher allele frequency, and collection through methods other than clinical testing. It is important to note that the reinterpretation of cancer predisposition genes requires a multidisciplinary effort involving clinicians, genetic counselors, bioinformaticians, and researchers [74].

In summary, the reinterpretation of cancer predisposition genes due to annotation inconsistencies among distinct database is an important issue that requires careful consideration and multidisciplinary collaboration. The use of updated annotation and rigid guideline follow-up and the integration of multiple types of genomic data can help improve the accuracy of cancer risk assessment and inform personalized prevention and treatment strategies.

### Strength and limitations

This study analyzing *BRCA1*, *BRCA2*, and *PALB2* mutations through tumor-targeted sequencing boosts several notable strengths. Firstly, it is the first large-scale analysis of its kind in Taiwan and Asia, involving a substantial number of breast cancer cases. Additionally, the study benefits from the availability of data on family history, molecular subtypes, and early or advanced breast cancer status. Moreover, by utilizing updated annotation databases and guidelines during interpretation and annotation, the study achieves enhanced comprehensiveness and accuracy in its results.

There are several limitations to consider. First, additional germline sequencing has been conducted with compromised recalled rates. Second, the sample size varied among different clinical groups, which may pose a challenge. Third, current study confined to only three genes, potentially narrowing the assessment of the genetic landscape. Lastly, a more in-depth evaluation of clinical outcomes and subgroup analyses, incorporating clinical-pathological factors and treatment regimens, is essential for a deeper understanding of Taiwanese breast cancer and should be conducted in future studies.

## Conclusion

In conclusion, our study reported a cohort of Taiwanese breast cancers harboring mutations in *BRCA1*, *BRCA2*, and *PALB2* through tumor-only sequencing, which underscores the impact of *BRCA1/2* and *PALB2* on breast cancer risk and potential therapeutic opportunities. Tumor-only sequencing has enabled a greater number of patients to uncover their genomic alterations, which offers additional insights for management strategies. These include recommendations for germline testing and the prospective utilization of PARP inhibitors to augment treatment efficacy. Nonetheless, supplementary germline testing remains critical, and investigating alternative methods for distinguishing whether variants are germline or somatic origin are invaluable.

### Supplementary Information

**Additional file 1. Table S1**. The result of all the variants from reflex germline testing.

## Data Availability

The datasets used and analyzed during the current study are available from the corresponding author on reasonable request.

## Abbreviations

HR: Homologous recombination
PARP inhibitors: Poly ADP-ribose polymerase inhibitors
g*BRCA1/2*: Germline *BRCA1/2*
s*BRCA1/2*: Somatic *BRCA1/2*
ORR: Objective response rate
TNBC: Triple-negative breast cancer
SDM: Share-decision making
IHC: Immunohistochemistry
ER: Estrogen receptor
PR: Progesterone receptor
HER2: Human epidermal growth factor receptor II
NGS: Next-generation sequencing
SNVs: Single nucleotide variants
CNVs: Copy number variations
TMO: Thermo Fisher
AA: Amino acid
P/LP: Pathogenic/Likely pathogenic
GGI: Gene–gene interaction
NSCLC: Non-Small Cell Lung Cancer
CGT: Clinical germline testing
VUS: Variants of uncertain significance
WES: Whole exome sequencing
WGS: Whole genome sequencing
LOHGIC: LOH-germline inference calculator
SGZ: Somatic-germline-zygosity
