# Supplementary material for: Prevalence of BRCA1, BRCA2, and PALB2 genomic alterations among 924 Taiwanese breast cancer assays with tumor-only targeted sequencing: extended data analysis from the VGH-TAYLOR study
Source: Breast Cancer Res. 2023 Dec 14;25:152. doi: 10.1186/s13058-023-01751-z (PMC10722686; doi:10.1186/s13058-023-01751-z)
Supplement: Supplementary file 1 — Additional file 1. Table S1. The result of all the variants from reflex germline testing. [file 13058_2023_1751_MOESM1_ESM.docx]

|  | | Gene status | | | | | | | | | | | | | | | | | | | |
| --- | --- | --- | --- | --- | --- | --- | --- | --- | --- | --- | --- | --- | --- | --- | --- | --- | --- | --- | --- | --- | --- |
|  |  | Not mutated | | Mutated | | p Value | *BRCA1* (-) | | *BRCA1* (+) | | p Value | *BRCA2* (-) | | *BRCA2* (+) | | p Value | *PALB2* (-) | | *PALB2* (+) | | p Value |
|  |  | N | % | N | % |  | N | % | N | % |  | N | % | N | % |  | N | % | N | % |  |
| **Total (N=879)** | | **749** | **85.2%** | **130** | **14.8%** | - | **852** | **96.9%** | **27** | **3.1%** | - | **803** | **91.4%** | **76** | **8.6%** | - | **833** | **94.8%** | **46** | **5.2%** | - |
| Group | Retro | 48 | 87.3% | 7 | 12.7% | 0.069 | 53 | 96.4% | 2 | 3.6% | **0.002** | 50 | 90.9 | 5 | 9.1% | 0.454 | 51 | 92.7% | 4 | 7.3% | 0.604 |
|  | Group 1A | 506 | 87.5% | 72 | 12.5% |  | 569 | 98.4% | 9 | 1.6% |  | 535 | 92.6% | 43 | 7.4% |  | 533 | 95.7% | 25 | 4.3% |  |
|  | Group 1B | 18 | 81.8% | 4 | 18.2% |  | 21 | 95.5% | 1 | 4.5% |  | 19 | 86.4% | 3 | 13.6% |  | 21 | 95.5% | 1 | 4.5% |  |
|  | Group 2 | 91 | 77.8% | 26 | 22.2% |  | 111 | 94.9% | 6 | 5.1% |  | 102 | 87.2% | 15 | 12.8% |  | 109 | 93.2% | 8 | 6.8% |  |
|  | Group 3-1 | 33 | 82.5% | 7 | 17.5% |  | 38 | 95.0% | 2 | 5.0% |  | 37 | 92.5% | 3 | 7.5% |  | 36 | 90.0% | 4 | 10.0% |  |
|  | Group 3-2 | 53 | 79.1% | 14 | 20.9% |  | 60 | 89.6% | 7 | 10.4% |  | 60 | 89.6% | 7 | 10.4% |  | 63 | 94.0% | 4 | 6.0% |  |
| Stage | EBC | 597 | 85.9% | 98 | 14.1% | 0.264 | 680 | 97.8% | 15 | 2.2% | **0.002** | 637 | 91.7% | 58 | 8.3% | 0.537 | 662 | 95.3% | 33 | 4.7% | 0.475 |
|  | ABC | 152 | 82.6% | 32 | 17.4% |  | 172 | 93.5% | 12 | 6.5% |  | 166 | 90.2% | 18 | 9.8% |  | 171 | 92.9% | 13 | 7.1% |  |
| Subtype | HR+/HER2- | 482 | 85.2% | 84 | 14.8% | 0.713 | 553 | 97.7% | 13 | 2.3% | 0.090 | 515 | 91.0% | 51 | 9.0% | 0.921 | 536 | 94.7% | 30 | 5.3% | 0.934 |
|  | HR+/HER2+ | 78 | 85.7% | 13 | 14.3% |  | 88 | 96.7% | 3 | 3.3% |  | 82 | 90.1% | 9 | 9.9% |  | 87 | 95.6% | 4 | 4.4% |  |
|  | HR-/HER2+ | 67 | 88.2% | 9 | 11.8% |  | 74 | 97.4% | 2 | 2.6% |  | 70 | 92.1% | 6 | 7.9% |  | 73 | 96.1% | 3 | 3.9% |  |
|  | TNBC | 108 | 82.4% | 23 | 17.6% |  | 122 | 93.1% | 9 | 6.9% |  | 122 | 93.1% | 9 | 6.9% |  | 122 | 93.1% | 9 | 6.9% |  |
|  | No data | 14 | 93.3% | 1 | 6.7% |  | 15 | 100% | 0 | 0% |  | 14 | 93.3% | 1 | 6.7% |  | 15 | 100.0% | 0 | 0.0% |  |
| Family history, BC | No BC family history | 550 | 85.8% | 91 | 14.2% | 0.719 | 622 | 97.0% | 19 | 3.0% | 0.920 | 590 | 92.0% | 51 | 8.0% | 0.204 | 609 | 95.5% | 32 | 5.0% | 0.891 |
|  | BC family history (+) | 133 | 83.6% | 26 | 16.4% |  | 154 | 96.9% | 5 | 3.0% |  | 145 | 91.2% | 14 | 8.8% |  | 149 | 93.7% | 10 | 6.3% |  |
|  | No data | 66 | 83.5% | 13 | 16.5% |  | 76 | 96.2% | 3 | 3.8% |  | 68 | 86.1% | 11 | 13.9% |  | 75 | 94.9% | 4 | 5.1% |  |
| Family history, OC | No OC family history | 671 | 85.9% | 110 | 14.1% | **0.002** | 758 | 97.1% | 23 | 2.9% | 0.679 | 720 | 92.2% | 61 | 7.8% | **0.011** | 741 | 94.9% | 40 | 5.1% | 0.857 |
|  | OC family history (+) | 8 | 53.3% | 7 | 46.7% |  | 14 | 93.3% | 1 | 6.7% |  | 11 | 73.3% | 4 | 26.7% |  | 13 | 86.7% | 2 | 13.3% |  |
|  | No data | 70 | 84.3% | 13 | 15.7% |  | 80 | 96.4% | 3 | 3.6% |  | 72 | 86.7% | 11 | 13.3% |  | 79 | 95.2% | 4 | 4.8% |  |

Table 1. Characteristic, all, BRCA1, BRCA2, PALB2, stratified by patients. EBC: early breast cancer, ABC: advanced breast cancer, HR: hormone receptor, HER2: human epidermal growth factor receptor 2; TNBC: triple-negative breasrt cancer, OC: ovarian cancer.

| A | B | Neither | A Not B | B Not A | Both | Log2 Odds Ratio | p-Value | q-Value | Tendency |
| --- | --- | --- | --- | --- | --- | --- | --- | --- | --- |
| *BRCA1* | *BRCA2* | 851 | 15 | 28 | 13 | >3 | **<0.001** | **<0.001** | Co-occurrence |
| *BRCA1* | *PALB2* | 843 | 20 | 38 | 8 | >3 | **<0.001** | **<0.001** | Co-occurrence |
| *BRCA2* | *PALB2* | 828 | 33 | 38 | 8 | 2.401 | **<0.001** | **<0.001** | Co-occurrence |

Table 2. Mutual exclusivity analysis of *BRCA1*, *BRCA2*, *PALB2* mutations among assayed patients.

Note: 17 samples were discarded for mutual exclusivity due to missing value in at least one of the interrogated genes.

| Case no. | No. of genes | The list of genes | No. of variants | The list of variants |
| --- | --- | --- | --- | --- |
| 1 | 2 | *BRCA1*  *PALB2* | 2 | *BRCA1* p.(E1257fs) c.3770_3771delAG  *PALB2* p.(I887fs) c.2659_2660delAT |
| 2 | 1 | *BRCA2* | 2 | *BRCA2* p.(E1571fs) c.4712_4713delAG  *BRCA2* p.(N372H) c.1114A>C |
| 3 | 1 | *PALB2* | 2 | *PALB2* p.(F1181fs) c.3540_3541delAT  *PALB2* p.(V870fs) c.2607delC |
| 4 | 2 | *BRCA2*  *PALB2* | 2 | *BRCA2* p.(V2503fs) c.7506_7507insA  *PALB2* p.(I887fs) c.2659_2660delAT |
| 5 | 2 | *BRCA1*  *PALB2* | 2 | *BRCA1* p.(K654fs) c.1960_1961insG  *PALB2* p.(M723fs) c.2167_2168delAT |
| 6 | 1 | *PALB2* | 2 | \| *PALB2* p.(P713fs) c.2138delC \| \| --- \| \| *PALB2* p.(Y743*) c.2229T>A \| |
| 7 | 1 | *BRCA2* | 2 | *BRCA2 p.(Q2499*) c.7495C>T*  *BRCA2* c.476-3C>T |
| 8 | 2 | *BRCA1*  *BRCA2* | 2 | *BRCA1* p.(R1720Q) c.5159G>A  *BRCA2* p.(W2970*) c.8910G>A |
| 9 | 1 | *BRCA2* | 2 | *BRCA2* p.(S2186fs) c.6556_6557insA  *BRCA2* p.(X159_splice) c.476-2A>G |
| 10 | 2 | *BRCA1*  *BRCA2* | 2 | *BRCA1* p.(S267fs) c.799_800insT  *BRCA2* p.(T912fs) c.2734_2735insA |
| 11 | 1 | *BRCA2* | 3 | *BRCA2* p.(L1635*) c.4904T>A  *BRCA2* p.(S2186fs) c.6556_6557insA  *BRCA2* p.(X159_splice) c.476-2A>G |
| 12 | 2 | *BRCA1*  *BRCA2* | 3 | **BRCA1* c.5137+1G>A  *BRCA2* p.(Q2539*) c.7615C>T  *BRCA2* p.(X2659_splice) c.7976+2C>T |
| 13 | 2 | *BRCA2*  *PALB2* | 4 | *BRCA2* p.(E1493fs) c.4477delG  *BRCA2* p.(Q3227*) c.9679C>T  *BRCA2* p.(W194*) c.582G>A  *PALB2* p.(E1018*) c.3052G>T |
| 14 | 2 | *BRCA1*  *BRCA2* | 5 | *BRCA1* p.(T1376fs) c.4126_4127insA  *BRCA2* p.(E33*) c.96_97insT  *BRCA2* p.(S3041fs) c.9121_9122insT  *BRCA2* p.(S3147fs) c.9439_9440insT  *BRCA2* p.(T912fs) c.2734_2735insA |
| 15 | 2 | *BRCA1*  *BRCA2* | 5 | *BRCA1* p.(Q855*) c.2563C>T  *BRCA1* p.(X1760_splice) c.5341-3C>T  *BRCA2* p.(Q1124*) c.3370C>T  *BRCA2* p.(Q126*) c.376C>T  *BRCA2* p.(Q3295*) c.9883C>T |
| 16 | 3 | *BRCA1*  *BRCA2*  *PALB2* | 5 | *BRCA1* p.(Q1867*) c.5599C>T  *BRCA2* p.(Q2829*) c.8485C>T  *BRCA2* p.(Q649*) c.1945C>T  *BRCA2* c.6842-1G>A  *PALB2* c.2586+1G>A |
| 17 | 3 | *BRCA1*  *BRCA2*  *PALB2* | 7 | *BRCA1* p.(X27_splice) c.80+1G>A  *BRCA1* p.(Q1577*) c.4729C>T  *BRCA2* p.(R3052Q) c.9155G>A  *BRCA2* p.(R2520*) c.7558C>T  *PALB2* p.(Q141*) c.421C>T  *PALB2* p.(Q228*) c.682C>T  *PALB2* p.(Q921*) c.2761C>T |
| 18 | 3 | *BRCA1*  *BRCA2*  *PALB2* | 8 | *BRCA1* p.(S1180fs) c.3538_3539insA  *BRCA2* p.(S538fs) c.1612_1613insA  *BRCA2* p.(S973fs) c.2916_2917insA  *BRCA2* p.(T598fs) c.1792_1793insA  *PALB2* p.(D1125fs) c.3372_3373insA  *PALB2* p.(N342fs) c.1025_1026insA  *PALB2* p.(N368fs) c.1103_1104insA  *PALB2* p.(S357fs) c.1068_1069insA |
| 19 | 2 | *BRCA1*  *BRCA2* | 10 | *BRCA1* p.(Q1227*) c.3679C>T  *BRCA1* p.(Q1625*) c.4873C>T  *BRCA1* p.(Q1867*) c.5599C>T  *BRCA1* p.(W1739*) c.5216G>A  *BRCA1* p.(W1739*) c.5217G>A  *BRCA2* p.(X2985_splice) c.8953+1G>A  *BRCA2* p.(D2723N) c.8167G>A  *BRCA2* p.(Q66*) c.196C>T  *BRCA2* p.(W3191*) c.9572G>A  *BRCA2* p.(W993*) c.2978G>A |
| 20 | 2 | *BRCA1*  *BRCA2* | 15 | *BRCA1* p.(X1366_splice) c.4096+1G>A  *BRCA1* p.(X1559_splice) c.4738+1G>A  *BRCA1* p.(X183_splice) c.547+1G>A  *BRCA1* p.(W321*) c.963G>A  *BRCA2* p.(X106_splice) c.317-3C>T  *BRCA2* p.(G2313D) c.6938G>A  *BRCA2* p.(X2602_splice) c.7806-3C>T  *BRCA2* p.(E2220fs) c.6658delG  *BRCA2* p.(Q2100*) c.6298C>T  *BRCA2* p.(Q2491*) c.7471C>T  *BRCA2* p.(Q2506*) c.7516C>T  *BRCA2* p.(Q2823*) c.8467C>T  *BRCA2* p.(Q66*) c.196C>T  *BRCA2* p.(R2494*) c.7480C>T  *BRCA2* p.(W2990*) c.8969G>A |
| 21 | 3 | *BRCA1*  *BRCA2*  *PALB2* | 16 | *BRCA1* p.(Q1135*) c.3403C>T  *BRCA1* p.(Q538*) c.1612C>T  *BRCA1* p.(Q905*) c.2713C>T  *BRCA1* p.(T1706I) c.5117C>T  *BRCA1* p.(W385*) c.1155G>A  *BRCA2* p.(X2602_splice) c.7805+1G>A  *BRCA2* p.(Q1379*) c.4135C>T  *BRCA2* p.(Q1623*) c.4867C>T  *BRCA2* p.(Q2823*) c.8467C>T  *BRCA2* p.(R2659K) c.7976G>A  *BRCA2* p.(W194*) c.581G>A  *BRCA2* p.(W2574*) c.7722G>A  *BRCA2* p.(W2586*) c.7758G>A  *BRCA2* p.(W2725*) c.8174G>A  *PALB2* p.(Q1023*) c.3067C>T  *PALB2* p.(Q856*) c.2566C>T |
| 22 | 3 | *BRCA1*  *BRCA2*  *PALB2* | 19 | *BRCA1* p.(X71_splice) c.212+1G>A  *BRCA1* c.4096+1G>A c.4096+1G>A  *&*BRCA1* c.5215+1G>A  *&*BRCA1* c.5256+1G>A  *BRCA1* p.(X183_splice) c.547+1G>A  *BRCA1* p.(A1729V) c.5186C>T  *BRCA1* p.(W1836*) c.5507G>A  *BRCA2* p.(X142_splice) c.425+1G>A  *BRCA2* c.8488-1G>A c.8488-1G>A  *BRCA2* p.(X3217_splice) c.9649-3C>T  *BRCA2* p.(Q1138*) c.3412C>T  *BRCA2* p.(Q2024*) c.6070C>T  *BRCA2* p.(W2626*) c.7878G>A  *PALB2* p.(X839_splice) c.2515-3C>T  *PALB2* p.(X1038_splice) c.3114-1G>A  *PALB2* p.(X1117_splice) c.3351-3C>T  *PALB2* p.(E1002fs) c.3004delG  *PALB2* p.(Q370*) c.1108C>T  *PALB2* p.(Q568*) c.1702C>T |
| 23 | 3 | *BRCA1*  *BRCA2*  *PALB2* | 24 | *BRCA1* p.(K1780fs) c.5339_5340insA  *BRCA1* p.(N1542fs) c.4625_4626insA  *BRCA1* p.(Q1096fs) c.3285_3286insA  *BRCA1* p.(Q94fs) c.279_280insT  *BRCA1* p.(R1012fs) c.3034_3035insA  *BRCA1* p.(S267fs) c.799_800insT  *BRCA1* p.(T1376fs) c.4126_4127insA  *BRCA1* p.(V409fs) c.1224_1225insA  *BRCA2* p.(E33*) c.96_97insT  *BRCA2* p.(L446fs) c.1337_1338insT  *BRCA2* p.(Q2499fs) c.7494_7495insA  *BRCA2* p.(Q2941fs) c.8820_8821insA  *BRCA2* p.(Q937fs) c.2808_2809insA  *BRCA2* p.(R645fs) c.1933_1934insA  *BRCA2* p.(S2056fs) c.6164_6165insT  *BRCA2* p.(S2976fs) c.8926_8927insA  *BRCA2* p.(T1483fs) c.4447_4448insA  *BRCA2* p.(T1858fs) c.5572_5573insA  *BRCA2* p.(T2197fs) c.6589_6590insA  %*BRCA2* p.(T441fs) c.1317delT, c.1320_1321insT  *BRCA2* p.(T912fs) c.2734_2735insA  *PALB2* p.(E1002fs) c.3003_3004insA  *PALB2* p.(L58fs) c.173_174insT  *PALB2* p.(T841fs) c.2521_2522insA |
| 24 | 3 | *BRCA1*  *BRCA2*  *PALB2* | 25 | *BRCA1* p.(E730fs) c.2187_2188insA  *BRCA1* p.(H1402fs) c.4203_4204insA  *BRCA1* p.(I1108fs) c.3322_3323insA  *BRCA1* p.(N1018fs) c.3053_3054insA  *BRCA1* p.(N1309fs) c.3926_3927insA  *BRCA1* p.(P371fs) c.1110_1111insT  *BRCA1* p.(P684fs) c.2048_2049insA  *BRCA1* p.(Q1096fs) c.3285_3286insA  *BRCA1* p.(Q1323fs) c.3966_3967insA  *BRCA2* p.(D2819*) c.8454_8455insT  *BRCA2* p.(D2900fs) c.8697_8698insA  *BRCA2* p.(E1593fs) c.4776_4777insA  *BRCA2* p.(E2301fs) c.6900_6901insA  *BRCA2* p.(E33*) c.96_97insT  *BRCA2* p.(L61fs) c.181_182insA  *BRCA2* p.(N3213fs) c.9638_9639insA  *BRCA2* p.(Q2941fs) c.8820_8821insA  *BRCA2* p.(Q937fs) c.2808_2809insA  *BRCA2* p.(S131fs) c.391_392insT  *BRCA2* p.(S2616fs) c.7846_7847insT  *BRCA2* p.(S3241fs) c.9721_9722insT  *BRCA2* p.(T1483fs) c.4447_4448insA  *BRCA2* p.(T2207fs) c.6619_6620insA  *PALB2* p.(A770fs) c.2307_2308insT  *PALB2* p.(L58fs) c.173_174insT |

Table 3. The list of multiple variants per gene or more than one mutant gene.

*No amino acid (AA) change was found.

&The novel variants.

%Two different codings resulted into the same amino acid (AA) change.

|  | **Gene** | **Site** | **Location** | **Transcript** | **Coding** | **Allele Frequency** | **ClinVAR** | **Oncomine** | **OncoKB™** |
| --- | --- | --- | --- | --- | --- | --- | --- | --- | --- |
| 1 | *BRCA1* | splicesite | chr17:41215349 | NM_007300.3 | c.5256+1G>A | 12.53% | Pathogenic | Not recorded | No reference |
| 2 | *BRCA1* | splicesite | chr17:41215890 | NM_007300.3 | c.5215+1G>A | 6.12% | Pathogenic | Hotspot | No reference |
| 3 | *BRCA2* | splicesite | chr13:32890556 | NM_000059.3 | c.-38-3CAG>C | 51.19% | Likely pathogenic | Not recorded | No reference |

Table 4. The list of novel variants discovered in the cohort.

|  | | No clinical implication | | With clinical implication | | Sum | p Value |
| --- | --- | --- | --- | --- | --- | --- | --- |
|  |  | N | % | N | % | N |  |
| Total | | 5 | 2.8% | 171 | 97.2% | 176 | - |
| Gene | *BRCA1* | 1 | 2.0% | 48 | 98.0% | 49 (27.8%) | 0.415 |
|  | *BRCA2* | 4 | 4.3% | 90 | 95.7% | 94 (53.4%) |  |
|  | *PALB2* | 0 | 0.0% | 33 | 100.0% | 33 (18.8%) |  |
| Site | exonic | 4 | 2.7% | 146 | 97.3% | 150 (85.2%) | 0.738 |
|  | splicesite | 1 | 3.8% | 25 | 96.2% | 26 (14.8%) |  |
| Type of mutation | frameshift deletion | 0 | 0.0% | 28 | 100.0% | 28 (15.9%) | **<0.001** |
|  | frameshift insertion | 0 | 0.0% | 52 | 100.0% | 52 (29.5%) |  |
|  | missense | **4** | **50.0%** | 4 | 50.0% | 8 (4.5%) |  |
|  | nonsense | 0 | 0.0% | 62 | 100.0% | 62 (35.2%) |  |
|  | unknown | 1 | 3.8% | 25 | 96.2% | 26 (14.8%) |  |
| Annotation, by Clinvar | Pathogenic | 0 | 0.0% | 68 | 100.0% | 68 (38.6%) | **<0.001** |
|  | Likely Pathogenic | 0 | 0.0% | 5 | 100.0% | 5 (2.8%) |  |
|  | Pathogenic/Likely Pathogenic | 0 | 0.0% | 6 | 100.0% | 6 (3.4%) |  |
|  | Conflicting | **2** | **50.0%** | 2 | 50.0% | 4 (2.3%) |  |
|  | Benign | 1 | 100.0% | 0 | 0.0% | 1 (0.6%) |  |
|  | Uncertain significance | 1 | 33.3% | 2 | 66.7% | 3 (1.7%) |  |
|  | unknown | 1 | 1.1% | 88 | 98.9% | 89 (50.6%) |  |
| Annotation, by Oncomine | No annotation | 3 | 13.6% | 19 | 86.4% | 22 (12.5%) | **<0.001** |
|  | Deleterious | 0 | 0.0% | **142** | **100.0%** | 142 (80.7%) |  |
|  | Hotspot | 2 | 16.7% | **10** | **83.3%** | 12 (6.8%) |  |
| Annotation, comparing ClinVar to Oncomine | ClinVar(+), Oncomine(+) | 0 | 0.0% | 70 | 100.0% | 70 (39.8%) | **<0.001** |
|  | ClinVar(+), Oncomine(-) | 0 | 0.0% | 9 | 100.0% | 9 (5.1%) |  |
|  | ClinVar(-), Oncomine(+) | 2 | 2.4% | 82 | 97.6% | 84 (47.7%) |  |
|  | ClinVar(-), Oncomine(-) | ***3*** | ***23.1%*** | 10 | 76.9% | 13 (7.4%) |  |

Table 5. Summary of amino acid changes in study cohort, stratified by evidence of clinical implication.

| **Items** | **N (cases)** | **%** |
| --- | --- | --- |
| Total cases | 130 | 100 |
| Cases that underwent whole genome sequencing (WGS) | 7 | 5.4 |
| Cases with pathogenic or likely pathogenic variants detected through tumor-only sequencing, planned for whole exome sequencing (WES) | 48 | 36.9 |
| Possible candidates for whole exome sequencing (WES) | 20 | 15.4 |
| Cases that underwent whole exome sequencing (WES) | 9 | 6.9 |
| Cases loss of follow up | 5 | 3.8 |
| Cases with no further clinical arrangements | 9 | 6.9 |
| Cases expired | 14 | 10.8 |
| Germline mutation detected, pathogenic variant | 4 | 3.1 |
| Germline mutation detected, uncertain significance | 2 | 1.5 |
| Germline mutation detected, benign | 5 | 3.8 |
| Pending result of whole genome sequencing (WGS) or whole exome sequencing (WES) | 5 | 3.8 |

Table 6. Characteristic of the cases undergoing reflex germline testing.

| **Categories** | **Total samples** | **%** | ***BRCA1*** | ***BRCA2*** | ***PALB2*** |
| --- | --- | --- | --- | --- | --- |
| Germline | 40 | 14.2 | 8 | 32 | 0 |
| Somatic | 169 | 60.1 | 50 | 96 | 22 |
| Borderline | 26 | 9.3 | 4 | 20 | 2 |
| No data | 46 | 16.4 | 3 | 2 | 41 |

Table 7. The results of the prediction of germline/somatic mutations by the simplified LOHGIC and SGZ method.

| Case no. | No. of genes | The list of genes | No. of variants | Annotation (ClinVar, OncoKB™) | The list of variants |
| --- | --- | --- | --- | --- | --- |
| 1 | 2 | *BRCA1*  *BRCA2* | 28 | Benign | \| *BRCA1* c.A4696G p.S1566G \| \| --- \| \| *BRCA1* c.A1525G p.S509G \| \| *BRCA1* c.A4837G p.S1613G \| \| *BRCA1* c.A1525G p.S509G \| \| *BRCA1* c.A4900G p.S1634G \| \| *BRCA1* c.T4167C p.S1389S \| \| *BRCA1* c.T999C p.S333S \| \| *BRCA1* c.T4308C p.S1436S \| \| *BRCA1* c.A3407G p.K1136R \| \| *BRCA1* c.A3548G p.K1183R \| \| *BRCA1* c.A2972G p.E991G \| \| *BRCA1* c.A3113G p.E1038G \| \| *BRCA1* c.A3113G p.E1038G \| \| *BRCA1* c.C2471T p.P824L \| \| *BRCA1* c.C2612T p.P871L \| \| *BRCA1* c.C2612T p.P871L \| \| *BRCA1* c.T2170C p.L724L \| \| *BRCA1* c.T2311C p.L771L \| \| *BRCA1* c.T2311C p.L771L \| \| *BRCA1* c.C1941T p.S647S \| \| *BRCA1* c.C2082T p.S694S \| \| *BRCA1* c.C2082T p.S694S \| \| *BRCA2* c.A3396G p.K1132K \| \| *BRCA2* c.T3807C p.V1269V \| \| *BRCA2* c.A4563G p.L1521L \| \| *BRCA2* c.G6513C p.V2171V \| \| *BRCA2* c.A7242G p.S2414S \| \| *BRCA2* c.T7397C p.V2466A \| |
| 2 | 2 | *BRCA1*  *BRCA2* | 28 | Benign | \| *BRCA1* c.A4696G p.S1566G \| \| --- \| \| *BRCA1* c.A1525G p.S509G \| \| *BRCA1* c.A4837G p.S1613G \| \| *BRCA1* c.A1525G p.S509G \| \| *BRCA1* c.A4900G p.S1634G \| \| *BRCA1* c.T999C p.S333S \| \| *BRCA1* c.T4308C p.S1436S \| \| *BRCA1* c.A3113G p.E1038G \| \| *BRCA1* c.A3113G p.E1038G \| \| *BRCA1* c.C2612T p.P871L \| \| *BRCA1* c.C2612T p.P871L \| \| *BRCA1* c.T2311C p.L771L \| \| *BRCA1* c.C2082T p.S694S \| \| *BRCA1* c.T2311C p.L771L \| \| *BRCA1* c.C2082T p.S694S \| \| *BRCA1* c.A3548G p.K1183R \| \| *BRCA1* c.T4167C p.S1389S \| \| *BRCA1* c.A3407G p.K1136R \| \| *BRCA1* c.A2972G p.E991G \| \| *BRCA1* c.C2471T p.P824L \| \| *BRCA1* c.T2170C p.L724L \| \| *BRCA1* c.C1941T p.S647S \| \| *BRCA2* c.A1114C p.N372H \| \| *BRCA2* c.A3396G p.K1132K \| \| *BRCA2* c.A4563G p.L1521L \| \| *BRCA2* c.G6513C p.V2171V \| \| *BRCA2* c.A7242G p.S2414S \| \| *BRCA2* c.T7397C p.V2466A \| |
| 3 | 3 | *BRCA1*  *BRCA2*  *PALB2* | 25 | Benign | \| *BRCA1* c.*1287C>T \| \| --- \| \| *BRCA1* c.*421G>T \| \| *BRCA1* c.*1287C>T \| \| *BRCA1* c.*421G>T \| \| *BRCA1* c.*1287C>T \| \| *BRCA1* c.*421G>T \| \| *BRCA1* c.*1393C>T \| \| *BRCA1* c.*527G>T \| \| *BRCA1* c.*1287C>T \| \| *BRCA1* c.*421G>T \| \| *BRCA2* c.A865C p.N289H \| \| *BRCA2* c.A1365G p.S455S \| \| *BRCA2* c.T2229C p.H743H \| \| *BRCA2* c.A2971G p.N991D \| \| *BRCA2* c.A3396G p.K1132K \| \| *BRCA2* c.A4563G p.L1521L \| \| *BRCA2* c.G6513C p.V2171V \| \| *BRCA2* c.A7242G p.S2414S \| \| *BRCA2* c.T7397C p.V2466A \| \| *BRCA2* c.T9069A p.A3023A \| \| *BRCA2* c.-26G>A \| \| *BRCA2* c.*532A>G \| \| *BRCA2* c.-26G>A \| \| *PALB2* c.A791G p.Q264R \| |
|  |  |  |  | Benign/likely benign | *PALB2* c.A1676G p.Q559R |
| 4 |  | *BRCA1* | 37 | Pathogenic | *BRCA1* c.3629_3630del p.E1210Gfs*9 |
|  |  | *BRCA2*  *PALB2* |  | Benign | \| BRCA1 c.A1411G p.S471G \| \| --- \| \| BRCA1 c.T4167C p.S1389S \| \| *BRCA1* c.A3548G p.K1183R \| \| *BRCA1* c.A3113G p.E1038G \| \| *BRCA1* c.C2612T p.P871L \| \| *BRCA1* c.T2566C p.Y856H \| \| *BRCA1* c.T2311C p.L771L \| \| *BRCA1* c.C2082T p.S694S \| \| *BRCA1* c.A4696G p.S1566G \| \| *BRCA1* c.T4308C p.S1436S \| \| *BRCA1* c.A3113G p.E1038G \| \| *BRCA1* c.C2612T p.P871L \| \| *BRCA1* c.T2566C p.Y856H \| \| *BRCA1* c.T2311C p.L771L \| \| *BRCA1* c.A4837G p.S1613G \| \| *BRCA1* c.T999C p.S333S \| \| *BRCA1* c.A1525G p.S509G \| \| *BRCA1* c.A3113G p.E1038G \| \| *BRCA1* c.C2612T p.P871L \| \| *BRCA1* c.T2566C p.Y856H \| \| *BRCA1* c.T2311C p.L771L \| \| *BRCA1* c.A1525G p.S509G \| \| *BRCA1* c.A310G p.S104G \| \| *BRCA1* c.T882C p.S294S \| \| *BRCA1* c.A3407G p.K1136R \| \| *BRCA1* c.A2972G p.E991G \| \| *BRCA1* c.C2471T p.P824L \| \| *BRCA1* c.T2425C p.Y809H \| \| *BRCA1* c.T2170C p.L724L \| \| *BRCA2* c.A3396G p.K1132K \| \| *BRCA2* c.G6513C p.V2171V \| \| *BRCA2* c.A7242G p.S2414S \| \| *BRCA2* c.T7397C p.V2466A \| \| *BRCA2* c.A1114C p.N372H \| \| *PALB2* c.A791G p.Q264R \| |
|  |  |  |  | Benign/likely benign | *PALB2* c.A1676G p.Q559R |
| 5 | 3 | *BRCA1*  *BRCA2*  *PALB2* | 6 | Benign | \| *BRCA2* c.A4563G p.L1521L \| \| --- \| \| *BRCA2* c.G6513C p.V2171V \| \| *BRCA2* c.T7397C p.V2466A \| \| *BRCA2* c.-806A>G \| \| *PALB2* c.*105A>C \| \| *PALB2* c.*369A>G \| |
| 6 | 2 | *BRCA1* | 9 | Pathogenic/likely pathogenic | *BRCA2* c.8755-1G>C |
|  |  | *BRCA2* |  | Benign | \| *BRCA1* c.T2425C p.Y809H \| \| --- \| \| *BRCA1* c.T2566C p.Y856H \| \| *BRCA2* c.*105A>C \| \| *BRCA2* c.T3807C p.V1269V \| \| *BRCA2* c.A4563G p.L1521L \| \| *BRCA2* c.G6513C p.V2171V \| \| *BRCA2* c.T7397C p.V2466A \| \| *BRCA2* c.A10234G p.I3412V \| |
| 7 | 1 | *BRCA2* | 7 | Uncertain significance | *BRCA2* c.A5624C p.K1875T |
|  |  |  |  | Benign | \| *BRCA2* c.A1114C p.N372H \| \| --- \| \| *BRCA2* c.A1362G p.K454K \| \| *BRCA2* c.A4563G p.L1521L \| \| *BRCA2* c.G6513C p.V2171V \| \| *BRCA2* c.T7397C p.V2466A \| \| *BRCA2* c.*105A>C \| |
| 8 | 2 | *BRCA1* | 8 | Pathogenic | *BRCA1* c.1969_1970del p.N657Cfs*7 |
|  |  | *BRCA2* |  | Benign | \| *BRCA2* c.A3396G p.K1132K \| \| --- \| \| *BRCA2* c.A4563G p.L1521L \| \| *BRCA2* c.G6513C p.V2171V \| \| *BRCA2* c.A7242G p.S2414S \| \| *BRCA2* c.-26G>A \| \| *BRCA2* c.*369A>G \| \| *BRCA2* c.T7397C p.V2466A \| |
| 9 | 2 | *BRCA2*  *PALB2* | 10 | Benign | \| *BRCA2* c.A865C p.N289H \| \| --- \| \| *BRCA2* c.A1365G p.S455S \| \| *BRCA2* c.T2229C p.H743H \| \| *BRCA2* c.A2971G p.N991D \| \| *BRCA2* c.T3807C p.V1269V \| \| *BRCA2* c.A4563G p.L1521L \| \| *BRCA2* c.G6513C p.V2171V \| \| *BRCA2* c.T7397C p.V2466A \| \| *BRCA2* c.-806A>G \| |
|  |  |  |  | Benign/likely benign | *PALB2* c.A1676G p.Q559R |
| 10 | 1 | *PALB2* | 1 | Likely pathogenic | *PALB2* c.448C>T |
| 11 | 1 | *BRCA2* | 1 | Uncertain significance | *BRCA2* c.561G>A |

Table S1. The result of all the variants from reflex germline testing.
